# Moonlighting role of meiotic SYCP1 in breast cancer: A chromatin-bound regulator of DNA repair, transcription, and drug resistance

**DOI:** 10.1126/sciadv.aea2067

**Published:** 2026-07-08

**Authors:** Louise C. Brennan, Oleg V. Grinchuk, Miguel Pachon-Penalba, Ieng F. Sou, Conor J. Fawcett, Claudia G. Nogueira, Megan Guthrie, Andrew D. Bates, Megan Hine, Amanda Thomaz, Andew B. Fielding, Owen R. Davies, Wee-Wei Tee, Urszula L. McClurg

**Affiliations:** ^1^ Institute of Systems, Molecular and Integrative Biology, University of Liverpool, Crown Street, Liverpool, UK.; ^2^ Chromatin Dynamics and Disease Epigenetics Lab, Institute of Molecular and Cell Biology (IMCB), Agency for Science, Technology and Research (A*STAR), 61 Biopolis Drive, Proteos, Singapore 138673, Republic of Singapore.; ^3^ Centre for Cell Biology, Institute of Cell Biology, University of Edinburgh, Michael Swann Building, Max Born Crescent, Edinburgh, UK.; ^4^ Institute of Medical Biology, Polish Academy of Sciences, Tylna 3A, Lodz, Poland.; ^5^ Bio-Med-Chem Doctoral School of the University of Lodz and Lodz Institutes of the Polish Academy of Sciences, 90-237 Lodz, Poland.; ^6^ Division of Biomedical and Life Sciences, Faculty of Health and Medicine, Lancaster University, Lancaster, UK.; ^7^ Department of Physiology, Yong Loo Lin School of Medicine, National University of Singapore, Singapore 117593, Republic of Singapore.; ^8^ NUS Centre for Cancer Research, Yong Loo Lin School of Medicine, National University of Singapore, 14 Medical Drive, Singapore 117599, Republic of Singapore.

## Abstract

Maintenance of genome integrity is essential for cellular homeostasis, and its perturbation leads to tumorigenesis. Here, we uncover an unanticipated somatic role for the synaptonemal complex protein SYCP1, previously regarded as strictly meiosis specific, in a broad spectrum of human cancers including breast cancer. Through integrative genomic, proteomic, and functional analyses, we demonstrate that SYCP1 is aberrantly reexpressed in tumor cells, where it actively promotes DNA damage repair, cell cycle progression, and malignant growth. SYCP1 binds chromatin at regulatory elements and directly controls transcriptional programs governing genome maintenance, including key effectors such as *CCNB1*, *PCNA*, *RAD51C*, and *H2AX*. Loss of SYCP1 impairs DNA repair kinetics, attenuates tumor cell proliferation and migration, and increases sensitivity to chemotherapeutics cisplatin and gemcitabine. Mechanistically, SYCP1 coimmunoprecipitates with chromatin remodeling complexes and transcription factors SP1 and SP2 and modulates their genomic occupancy and oncogenic transcriptional outputs. Clinically, high SYCP1 expression stratifies patients with poor prognosis and therapy resistance across multiple cancer types. Our findings illuminate a previously unrecognized moonlighting function of SYCP1 in somatic cancer cells and position it as a critical chromatin-associated regulator of genome stability, with implications for biomarker development and therapeutic targeting.

## Teaser

Cancer cells reactivate the meiotic protein SYCP1 to regulate chromatin, enhance DNA repair, and drive malignancy.

## INTRODUCTION

Preserving genome integrity is fundamental to the homeostasis and survival of somatic cells. Genomic instability is a defining hallmark of cancer and a major contributor to malignant transformation and therapeutic resistance (*1*). Similarly, genome stability is indispensable during gametogenesis, where meiosis imposes highly orchestrated chromosomal movements to ensure accurate homolog pairing and recombination. Unlike mitosis, meiosis introduces programmed DNA double-strand breaks (DSBs) to facilitate homologous recombination and crossover formation—processes that are essential for genetic diversity and proper chromosome segregation. This added layer of complexity is stabilized by the synaptonemal complex (SC), a supramolecular protein scaffold that mediates synapsis and promotes crossover maturation (*2*). Perturbations in SC assembly cause infertility (*3*).

In stark contrast, somatic cells must avoid recombination instability. The presence of haploid-like gene expression or unregulated DNA breakage in somatic tissues has deleterious consequences, causing chromosomal instability and accelerating tumorigenesis (*4*). To preserve genomic integrity, meiotic genes—particularly those involved in chromosome structure and recombination—are stringently silenced in somatic lineages. The human SC comprises eight autosomally encoded proteins—SYCP1, SYCP2, SYCP3, SYCE1, SYCE2, SYCE3, TEX12, and SIX6OS1—all canonically restricted to germ cells (*2*). However, recent studies reveal aberrant expression of SC components in diverse human cancers, classifying them among cancer-testis antigens (CTAs) or more broadly as germ cell cancer genes (GCCGs) (*4*–*9*). Given their pivotal roles in meiotic chromosome dynamics, the abnormal expression of SC proteins in somatic cancer cells represents a profound threat to genome stability, potentially fueling malignant evolution.

Cancer cells frequently co-opt developmental programs to achieve proliferative plasticity and survive environmental stressors. The abnormal expression of meiotic genes exemplifies this strategy, allowing tumors to exploit nonsomatic machineries for adaptive advantage. In this context, we identify SYCP1, the central transverse filament of the SC (*10*), to be aberrantly expressed in cancer with direct oncogenic potential. SYCP1 is repurposed in cancer cells to promote DNA repair, sustain proliferation, and drive resistance to genotoxic therapies.

Our data reveal that SYCP1 expression is a functional mediator of cancer cell fitness. In tumors, SYCP1 facilitates DNA damage repair and rewires transcriptional programs essential for cell cycle progression. Mechanistically, SYCP1 binds to gene promoters and chromatin regulatory regions, modulating the expression of critical DNA repair and replication factors, including *CCNB1*, *PCNA*, *RAD51C*, and *H2AX*. Loss of SYCP1 impairs these processes, abrogates cellular proliferation and migration, and sensitizes tumor cells to DNA-damaging agents gemcitabine and cisplatin.

Notably, SYCP1’s role in cancer diverges from its canonical function in meiosis. In the germ line, SYCP1 acts as a structural scaffold aligning homologous chromosomes to support recombination fidelity (*11*). In contrast, in the somatic context of cancer, SYCP1 operates as a chromatin-bound regulator of gene expression. We identify direct interactions between SYCP1 and chromatin remodeling factors and show that its loss disrupts the chromatin recruitment of transcriptional regulators SP1 and SP2. This suggests that SYCP1 is co-opted as a transcriptional effector, linking chromatin structure to oncogenic gene expression.

Because meiosis and mitosis operate in fundamentally distinct cellular environments—with divergent proteomes, epigenetic landscapes, and regulatory cues—meiotic proteins like SYCP1 may acquire previously unidentified functions when ectopically expressed in somatic cancer cells. Rather than merely reinstating meiotic processes, these proteins moonlight in oncogenic pathways, driving tumor progression through mechanisms distinct from their original biological context. Our findings uncover a previously unappreciated role for SYCP1 as a chromatin-associated modulator of genome stability in cancer, positioning it as both a mechanistic driver of tumor evolution within the cancer cell and a candidate therapeutic target.

## RESULTS

### SYCP1 is commonly aberrantly expressed in patients with cancer

Although prior studies have reported aberrant expression of *SYCP1* at the transcript level across various malignancies (*12*–*19*), the functional implications of this aberrant expression—and whether it results in detectable protein expression—have remained largely unresolved. To address this, we conducted a comprehensive analysis of *SYCP1* expression in patient-derived datasets and cancer cell lines.

**Fig. 1.**
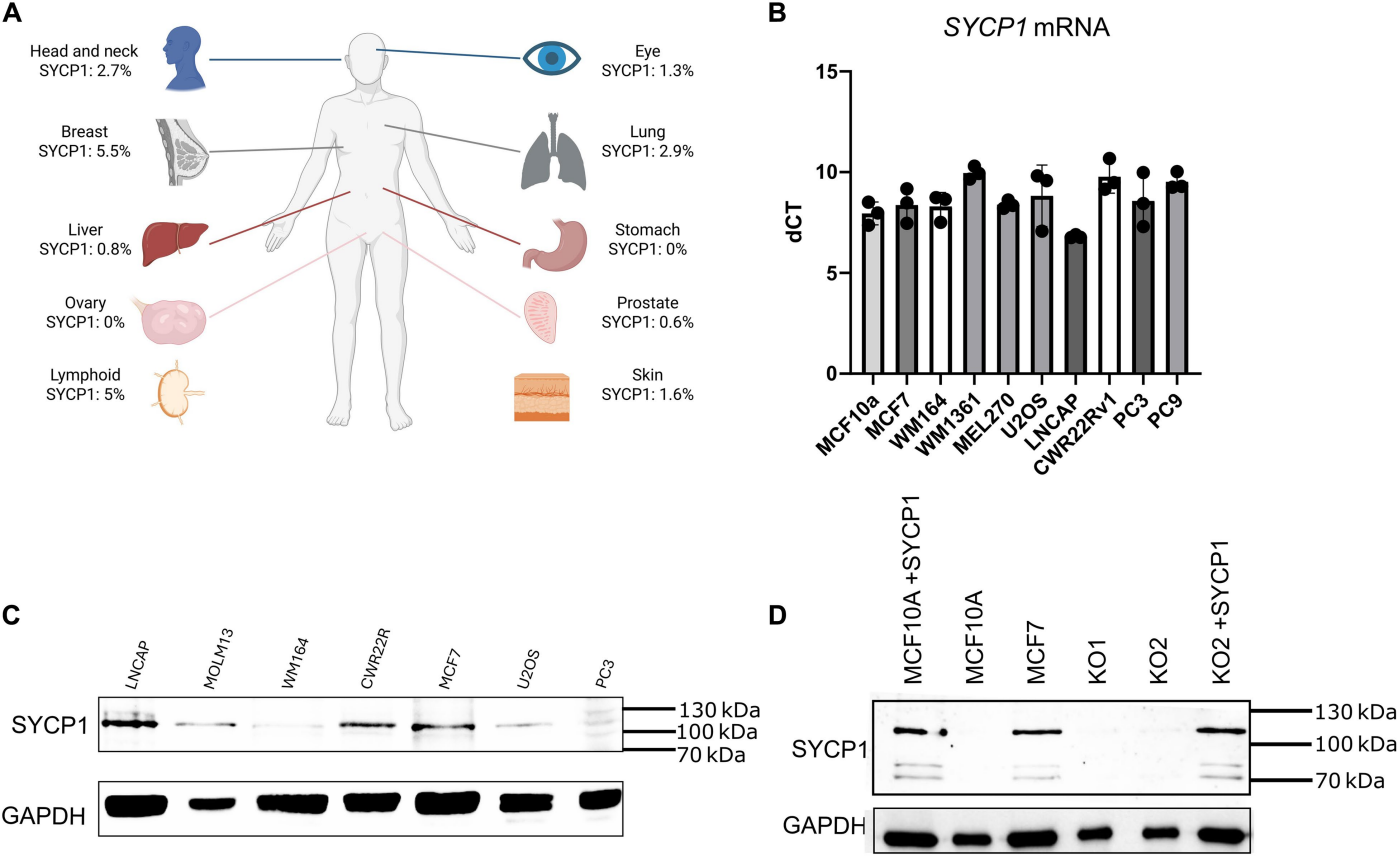
SYCP1 is commonly expressed in cancer. (**A**) Patients with cancer in TCGA were analyzed to determine % of patients with cancer with *SYCP1* mRNA expression in highlighted cancer subtypes by the organ of origin. Created in BioRender. McClurg, U. (2026) https://BioRender.com/kdxj9gk (**B**) *SYCP1* mRNA levels in cancer cell lines analyzed by qPCR and normalized to HPRT1. MCF10a nontransformed breast epithelium, MCF7 breast cancer, WM164 and WM1361 melanoma, Mel270 uveal melanoma, U2OS osteosarcoma, LNCAP, CWR22Rv1 and PC3 prostate cancer, and PC9 lung cancer. Error bars represent the SEM. (**C**) SYCP1 protein levels across cancer cell lines. LNCaP, PC3, and CWR22Rv1 prostate cancer, MCF7 breast cancer, U2OS osteosarcoma, WM164 melanoma, and MOLM13 AML. (**D**) SYCP1 protein levels in MCF7 breast cancer cells, two independent MCF7 SYCP1 KO clones, and MCF10A breast epithelial cells, including MCF10A and MCF7 KO cells transfected with SYCP1 (+SYCP1).

Systematic interrogation of The Cancer Genome Atlas (TCGA) revealed that *SYCP1* mRNA is aberrantly up-regulated across a wide array of solid tumors, with particularly high expression observed in lung and breast cancers. Notably, ~1 in 20 patients with these malignancies exhibited marked *SYCP1* mRNA overexpression (Fig. 1A). Elevated *SYCP1* expression significantly correlated with reduced overall survival in multiple cancer types, including cholangiocarcinoma, thymoma, skin cutaneous melanoma, uterine corpus endometrial carcinoma, and rectal and gastric adenocarcinomas (fig. S1). A similar negative association was observed with relapse-free survival (fig. S2), suggesting that *SYCP1* expression may be considered a possible clinically relevant prognostic biomarker.

To experimentally validate these observations, we assessed *SYCP1* expression across a panel of commonly used cancer cell lines. Notably, many of the tested models displayed robust expression of *SYCP1* at both the transcript (Fig. 1B and fig. S3) and protein levels (Fig. 1C). We further noticed that SYCP1 exhibited a distinct electrophoretic mobility shift, consistent with posttranslational modification, suggesting that it might be phosphorylated within the cell. Among these, MCF7 breast cancer cells exhibited particularly high levels of SYCP1 protein, whereas no detectable expression was observed in the nontransformed MCF10A breast epithelial line (Fig. 1D). This was further supported by analysis of SYCP1 protein levels in four healthy females, where no SYCP1 protein expression was observed across all cell types despite the *SYCP1* transcript being detected in select cell populations within the tissue (table S1). These findings underscore the cancer-specific nature of SYCP1 expression and highlight MCF7 cells as a relevant model for mechanistic interrogation.

Our data also revealed a weak correlation between *SYCP1* mRNA and protein levels, suggesting posttranscriptional regulation. This phenomenon has been widely described in the literature for a number of genes (*20*, *21*). These observations emphasize the importance of directly assessing protein expression when evaluating the aberrant expression of meiotic genes in cancer. Overall, our findings demonstrate that SYCP1 is aberrantly expressed in different cancers, where it may contribute functionally to tumor progression

### SYCP1 binds to chromatin in cancer cells

**Fig. 2.**
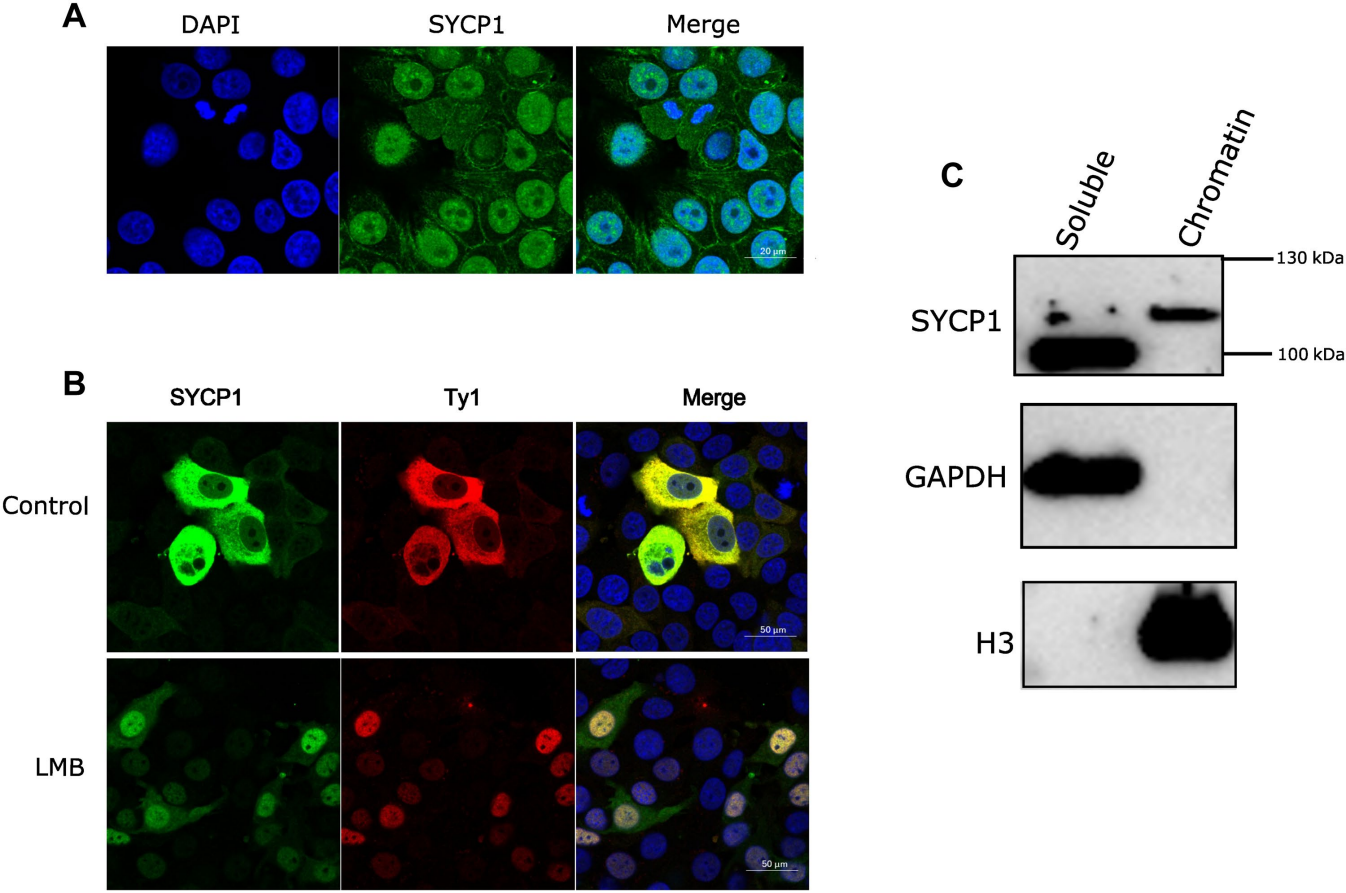
In cancer cells, SYCP1 localizes to the nucleus where it binds to the chromatin. (**A**) MCF7 cells with immunolabeled endogenous SYCP1. (**B**) MCF7 cells transfected with the Dox-inducible SYCP1-3xTy1 vector. SYCP1 overexpression was induced upon treatment with Dox (5 μg/ml). Where indicated, cells were treated with leptomycin B (LMB) for 3 hours. (**C**) MCF7 breast cancer cells were fractionated to chromatin and the soluble fraction containing the cytoplasm and nucleoplasm.

To elucidate the functional role of aberrantly expressed SYCP1 in cancer, we first examined its subcellular localization. Immunofluorescence analysis revealed that SYCP1 localizes predominantly to the nucleus with some low-level cytoplasmic immunolabeling in MCF7 breast cancer cells (Fig. 2A) and in additional cancer cell models (fig. S4, A to C). We also determined that SYCP1 nuclear localization was not affected by overexpression levels (fig. S4E). These findings were corroborated by biochemical cell fractionation, which confirmed nuclear enrichment of endogenous SYCP1 (fig. S4D).

To investigate the behavior of SYCP1 in a controlled system, we generated a doxycycline (Dox)–inducible SYCP1 construct fused to a 3xTy-1 epitope tag and established a stable MCF7 cell line expressing the tagged protein. Upon induction, SYCP1 localized to both the cytoplasm and the nucleus, with a pronounced cytoplasmic predominance under steady-state conditions. However, pharmacological inhibition of nuclear export resulted in exclusive nuclear retention of SYCP1 (Fig. 2B and fig. S4E), indicating that SYCP1 undergoes active nucleocytoplasmic shuttling in cancer cells.

To determine whether nuclear SYCP1 is associated with chromatin, we performed chromatin fractionation assays. These revealed that a substantial pool of endogenous SYCP1 is present in the chromatin-bound fraction, alongside a soluble nuclear fraction (Fig. 2C). Notably, the chromatin-associated form of SYCP1 exhibited a distinct electrophoretic mobility shift consistent with posttranslational modification, suggesting that chromatin binding may be regulated by specific molecular cues.

Together, these findings demonstrate that SYCP1 is not only expressed in cancer cells but also actively trafficked to the nucleus, where it engages with chromatin. This subcellular localization pattern supports a functional role for SYCP1 in regulating gene expression and chromatin dynamics in tumor cells.

### SYCP1 binds to the promoters of genes responsible for the DNA damage response and cell cycle

**Fig. 3.**
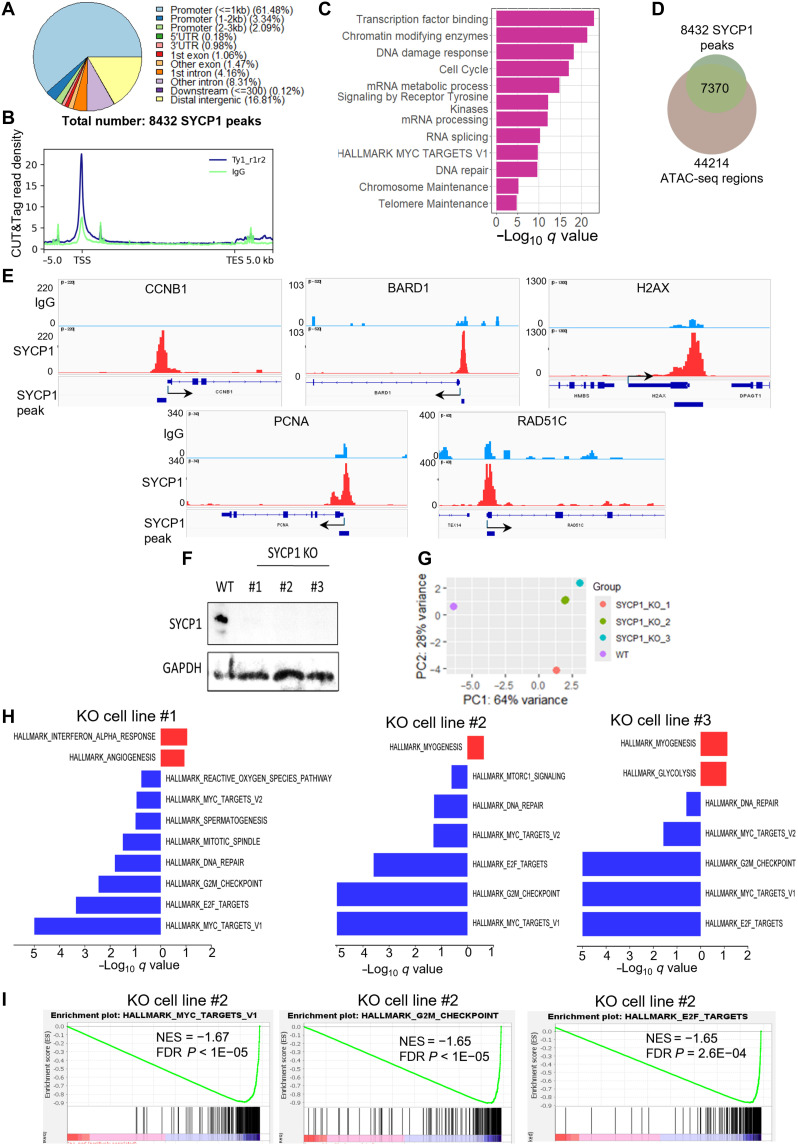
SYCP1 binds to the promoters of genes responsible for the DDR and cell cycle. (**A**) Total number and distribution of SYCP1 CUT&Tag DNA binding regions across genomic elements. (**B**) Metagene plot of SYCP1 binding levels within SYCP1-bound genes. The *y* axis represents the SYCP1 CUT&Tag read density, compared to the IgG negative control. TSS, transcription start site; TES, transcription end site. (**C**) Pathway enrichment analysis of genes associated with SYCP1-bound regions (bar plot). (**D**) SYCP1 CUT&Tag DNA binding regions are predominantly located in regions of open chromatin in MCF7 cells. (**E**) IGV browser view of selected CUT&Tag SYCP1 binding regions at the promoters of *CCNB1, BARD1, H2AX, PCNA*, and *RAD51C* genes. (**F**) SYCP1 protein levels in WT and CRISPR KO MCF7 lines. (**G**) Principal components analysis plot displaying that 64% of variance account for differences between WT samples (WT) and SYCP1 KO clones compared to only 34% of variance is responsible for differences among the KO clones. (**H** and **I**) Pathway enrichment analyses using the GSEAPreranked software (*39*, *40*) represented as a two-directional bar plot (H) or as a GSEA enrichment score plot (I). NES, normalized enrichment score.

To investigate the role of chromatin-bound SYCP1, we performed Cleavage Under Targets & Tagmentation (CUT&Tag) profiling (*22*) in the SYCP1-3xTy1 MCF7 Dox-inducible cell line, using a Ty1 targeting antibody to map SYCP1 genomic binding sites (Fig. 3). A total of 8432 CUT&Tag peaks were detected, and we found that SYCP1 predominantly binds to gene promoters (~68%; Fig. 3, A and B). Notably, SYCP1 was significantly enriched at promoters of genes responsible for transcription factor (TF) binding, RNA metabolism and processing, cell cycle, DNA repair, and response to stimuli (Fig. 3C). Of particular interest was SYCP1 localization to the promoters of key cell cycle genes (e.g., *CCNB1* and *PCNA*) (Fig. 3D) as well as genes involved in DNA damage response (DDR) (e.g., *BARD1*, *RAD51C*, and *H2AX*) (Fig. 3D) and DNA repair (*RECQL4*, *XPC*, and *OGG1*). Next, we examined whether SYCP1 DNA binding is associated with regions of open chromatin. Using publicly available ATAC-seq (assay for transposase-accessible chromatin using sequencing) data from MCF7 cells (*23*), we found that the vast majority of SYCP1-binding regions overlap with open chromatin (7870 of 8432 peaks) (Fig. 3D). However, these SYCP1-bound regions account for only ~17% of all open chromatin regions, indicating that SYCP1 binding is selective rather than random and suggesting that SYCP1 is recruited to a specific subset of accessible genomic regions (Fig. 3D). We observed strong enrichment of genes for receptor tyrosine kinases (e.g., *ERBB3*, *TRIB3*, and *GRB2*), suggesting a potential role for SYCP1 in modulating cancer-associated signaling pathways. In addition, SYCP1 showed abundant binding at the proximal promoters and/or gene bodies of ~30% of all histone genes (45 of 118 in the human genome), pointing to a possible role for SYCP1 in chromatin regulation.

### SYCP1 regulates transcription of genes involved in cell cycle control and DDR

To determine whether SYCP1 binding to gene promoters modulates transcriptional activity, we generated *SYCP1* knockout (KO) MCF7 cell lines using CRISPR-Cas9–mediated genome editing. As noted earlier, given the poor correlation between SYCP1 RNA and protein levels, we selected three independent KO clones based on confirmed loss of SYCP1 protein expression (Fig. 3E) and subjected them to transcriptomic profiling by RNA sequencing (RNA-seq).

Differential gene expression (DGE) analysis revealed a highly reproducible transcriptomic signature across all three *SYCP1* KO clones relative to wild-type (WT) MCF7 cells (Fig. 3F). Notably, the most significantly down-regulated pathways included cell cycle progression and DDR and repair mechanisms (Fig. 3, G and H). Key down-regulated genes included *CCNB1*, *CDC20*, *CDKN2C*, *CDKN2D*, *CENPA*, *H2AX*, *MCM4*, *MCM7*, *MYC*, *PCNA*, *POLD1*, *RAD51C*, *PLK4*, and *PRC1*—all of which are critical regulators of genomic maintenance and mitotic fidelity. Selected hits were validated by quantitative polymerase chain reaction (qPCR) (fig. S5). In contrast, *SYCP1* loss led to transcriptional up-regulation of genes associated with angiogenesis, glycolytic metabolism, and myogenic differentiation, such as *NFATC4*, *UVSSA*, *BMF*, *ATP6V1G1*, *TOM1*, and *ELAPOR1*.

**Fig. 4.**
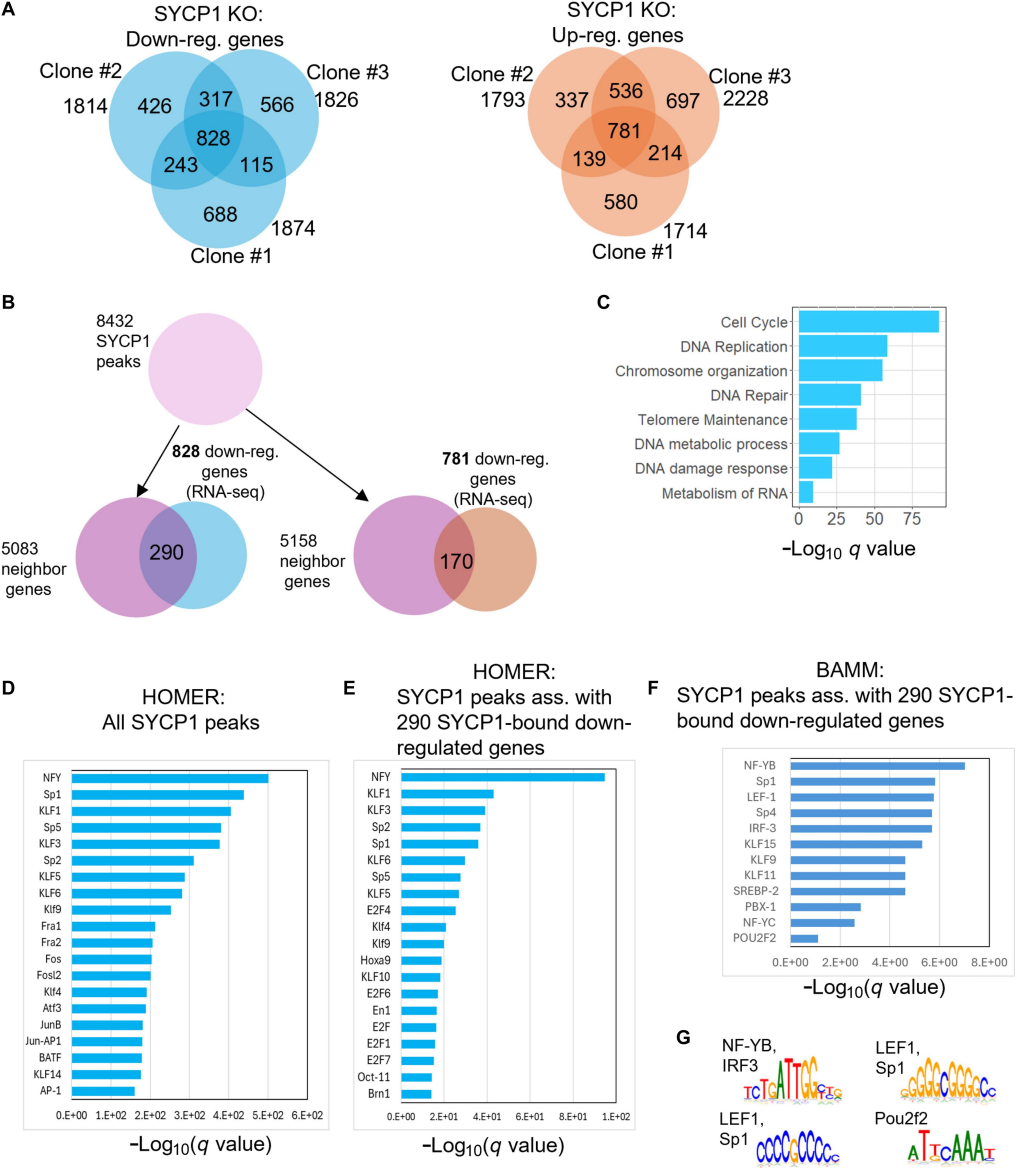
SYCP1 regulates expression of genes involved in the DDR and cell cycle. (**A** and **B**) Venn diagrams summarizing the results of RNA-seq analyses in three MCF7 SYCP1 KO cell lines. (**C**) Comparison of RNA-seq and CUT&Taq data analyses for SYCP1: The down-regulated overlapping gene list (290 genes) is strongly overenriched (1.37-fold, *P* = 2.2 × 10^−10^), and the up-regulated overlapping gene list (170 genes) is weakly underenriched (1.18-fold, *P* = 0.004), hypergeometric test; *q* value = 0.01. (**D**) Bar plot showing the TF enrichment analysis for all SYCP1 promoter-bound genes; *q* value = 1 × 10^−5^. TF enrichment analysis for 290 SYCP1 promoter-bound and SYCP1 KO down-regulated genes using (**E**) the HOMER software (http://homer.ucsd.edu/homer/) and (**F**) the BAMMmotif software (https://bammmotif.mpibpc.mpg.de/). (**G**) Enriched motifs with correspondingly assigned TFs are shown at the bottom.

To identify direct transcriptional targets of SYCP1, we integrated the transcriptomic data from the *SYCP1* KO clones (828 common down-regulated and 781 common up-regulated genes) with SYCP1 chromatin occupancy profiles generated by CUT&Tag. This integrative analysis identified 290 genes that were both significantly down-regulated and had SYCP1 occupancy at their promoter regions (Fig. 4, A and B). In contrast, only 170 promoter-bound genes were significantly up-regulated upon *SYCP1* deletion. Hypergeometric testing revealed a significant overrepresentation of SYCP1-bound genes among the down-regulated targets, whereas the overlap among up-regulated genes was weakly underenriched. These findings suggest that SYCP1 primarily acts as a transcriptional activator at the promoters it binds, directly regulating a gene network enriched in cell cycle, DDR, and RNA metabolic processes (Fig. 4C). Of particular interest, histone gene clusters were prominently enriched among the direct SYCP1 targets (fig. S6), implying a potential role for SYCP1 in chromatin assembly or replication-associated transcription.

To further characterize the transcriptional regulatory landscape at SYCP1-bound loci, we performed TF motif enrichment analysis within ±150 base pairs (bp) of SYCP1 peak summits. Using two distinct computational pipelines—HOMER and BAMMmotif—we interrogated both the global SYCP1 binding landscape (Fig. 4D) and the subset of 290 direct target genes (Fig. 4, E and F). Motifs enriched in these regions were annotated to known TFs using curated TF binding databases. Both tools converged on a core cohort of TFs implicated in cell cycle regulation, chromatin remodeling, and DNA repair (Fig. 4G), indicating that SYCP1 may function cooperatively with established oncogenic TFs to control proliferation and genome maintenance programs.

Together, these results establish SYCP1 as a chromatin-associated regulator that directly activates genes essential for cancer cell proliferation and survival by modulating transcriptional programs governing the cell cycle and genome stability.

### SYCP1 promotes cancer cell proliferation and migration

**Fig. 5.**
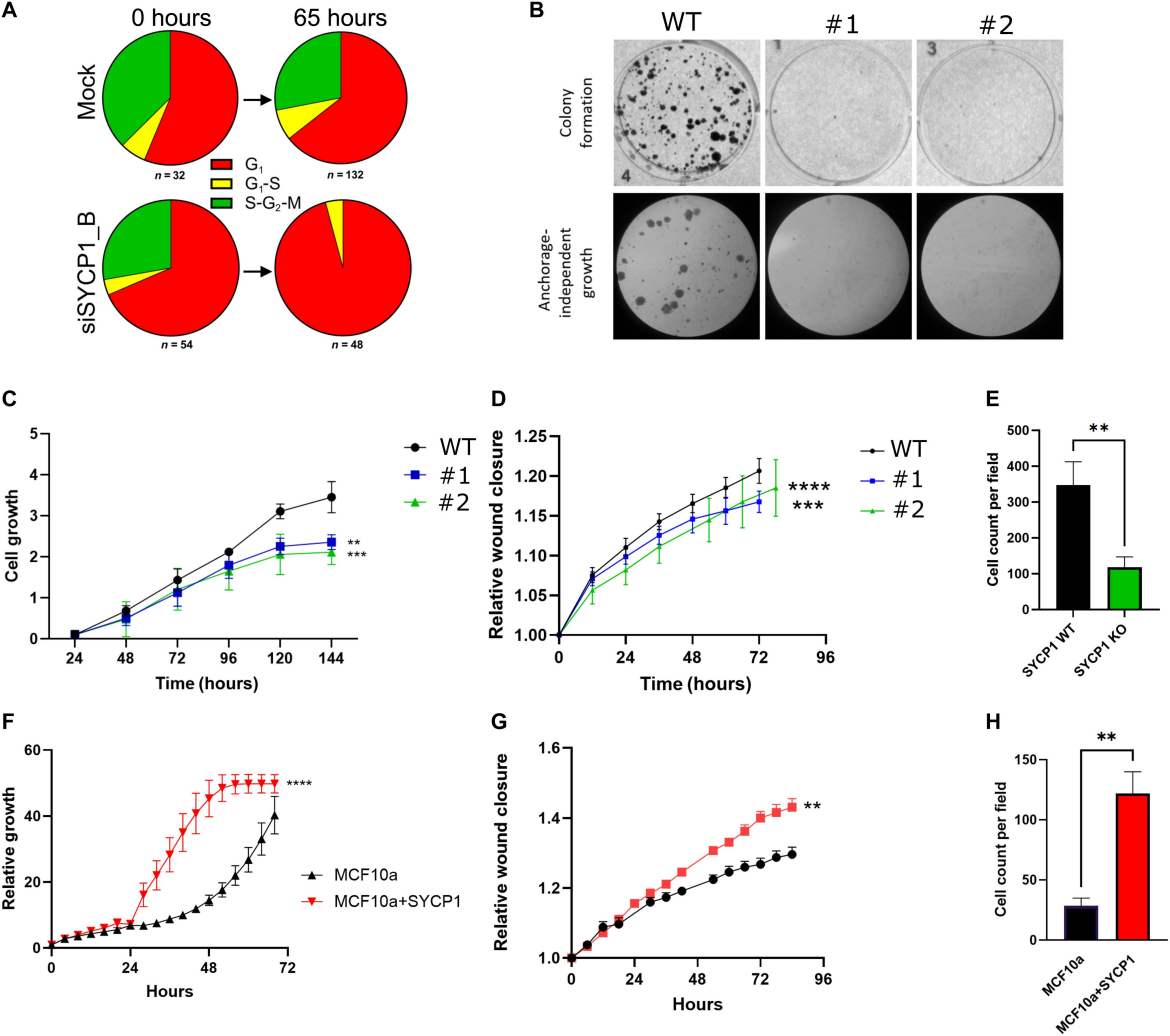
SYCP1 controls proliferation and migration of breast cancer cells. (**A**) MCF7 cells stably transfected with H2B-Fucci2a were transfected with mock control (SCR) or *SYCP1* targeting siRNAs. Twenty-four hours posttransfection, live cell imaging was initiated, with images acquired every 15 min for 65 hours. Pie charts show changes in cell cycle distribution. (**B**) WT and SYCP1 CRISPR KO lines of MCF7 cells were seeded at equal density on standard plates (colony formation) or in soft agar (anchorage-independent growth) and number of colonies after a week was compared. (**C**) WT and SYCP1 CRISPR KO lines of MCF7 cells were seeded at equal density, and cell numbers were tracked with Incucyte live cell imaging for 90 hours. (**D**) WT and SYCP1 CRISPR KO lines of MCF7 cells were seeded at equal density and scratched to create a wound with closure monitored by Incucyte live cell images for 90 hours. (**E**) WT and SYCP1 CRISPR KO lines of MCF7 cells were seeded at equal density into Transwells in serum-starved media with serum-rich media at the other side of the chamber. Following 12 hours of incubation, cells that migrated through the Transwell were counted. (**F**) WT and SYCP1 transfected MCF10A cells were seeded at equal density, and cell numbers were tracked with Incucyte live cell imaging for 90 hours. (**G**) WT and SYCP1 transfected MCF10A cells were seeded at equal density and scratched to create a wound with closure monitored by Incucyte live cell images for 90 hours. (**H**) WT and SYCP1 transfected MCF10A cells were seeded at equal density into Transwells in serum-starved media with serum-rich media at the other side of the chamber. Following 24 hours of incubation, cells that migrated through the Transwell were counted. Error bars represent the SEM in all figures.

Our transcriptomic and chromatin profiling analyses identified SYCP1 as a regulator of gene networks governing cell cycle progression and cell motility (Figs. 3 and 4). To directly assess the functional impact of SYCP1 depletion on cancer cell behavior, we first performed acute *SYCP1* knockdown (KD) in MCF7 breast cancer cells. Loss of SYCP1 resulted in a rapid and profound proliferative arrest, with abrogation of cell growth and G_1_ arrest in breast cancer cells observed within 72 hours postsilencing (Fig. 5A and fig. S7D). Similar growth inhibition phenotypes were observed in additional cancer models, underscoring a broader functional requirement for SYCP1 in tumor cells (fig. S7).

We next examined the long-term effects of complete SYCP1 ablation by analyzing stable *SYCP1* CRISPR KO clones. The markedly reduced efficiency in isolating viable KO clones highlighted a dependency of MCF7 cells on SYCP1 for sustained proliferation. Once established, *SYCP1*-null clones exhibited significantly impaired diminished clonogenic potential (Fig. 5), diminished proliferative capacity (Fig. 5C), and reduced migratory behavior, as assessed by both Transwell and scratch-wound assays (Fig. 5, D and E).

To determine whether SYCP1 expression alone is sufficient to induce a transformed phenotype, we ectopically expressed SYCP1 in nontumorigenic MCF10A breast epithelial cells. SYCP1 expression drove a marked increase in cell proliferation, with a >20-fold enhancement in viable cell number relative to vector control (Fig. 5F). Similarly, SYCP1-expressing MCF10A cells displayed enhanced migratory potential in both directed and collective migration assays (Fig. 5, G and H). Together, these findings reveal that SYCP1 is both necessary and sufficient to drive key tumorigenic properties, including uncontrolled proliferation and enhanced motility.

### DNA binding of SYCP1 is critical for its function

**Fig. 6.**
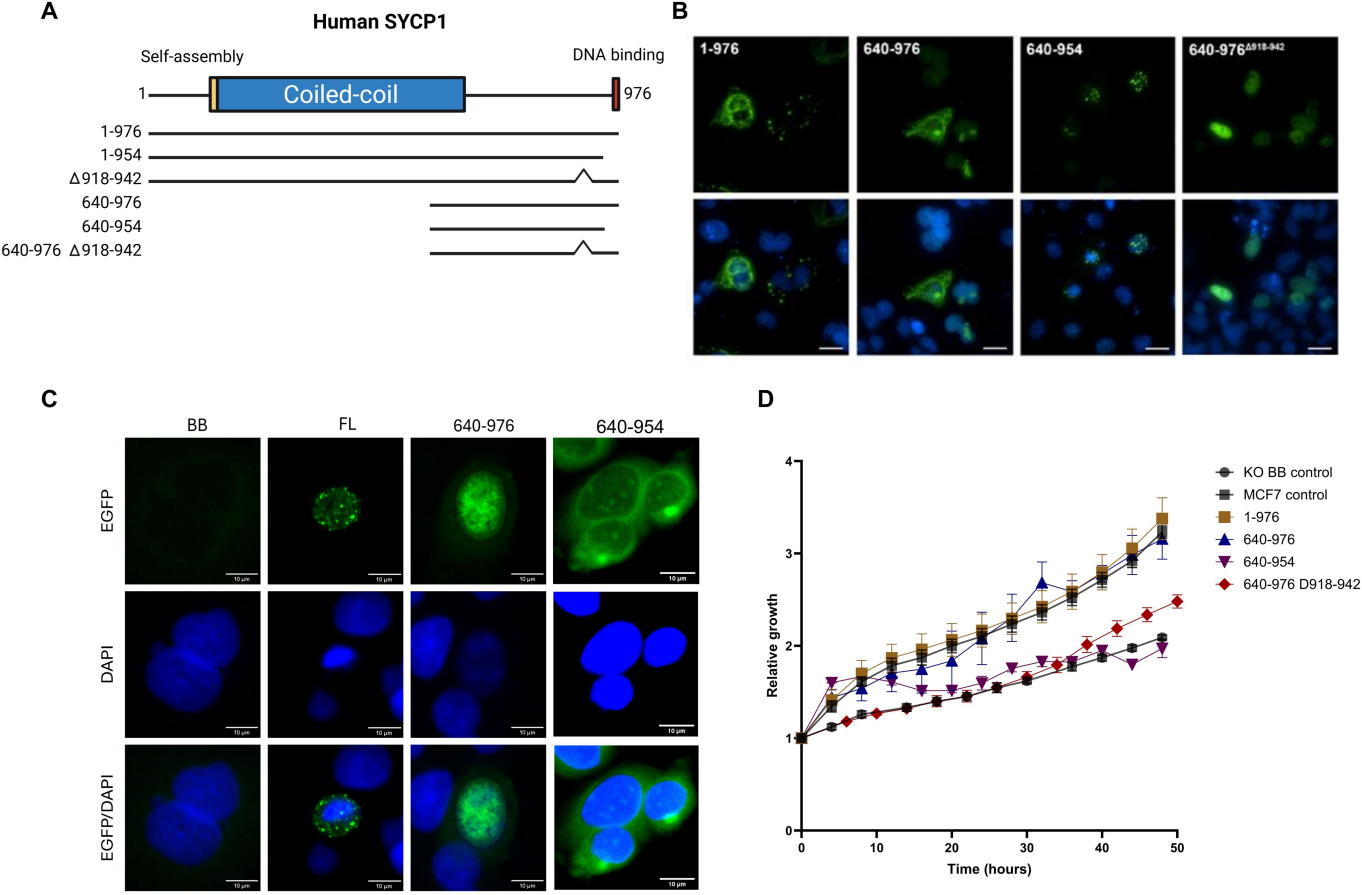
DNA binding of SYCP1 is critical for its function. (**A**) Structure of human SYCP1 and mutants used in this study. (**B**) Transient overexpression of EGFP-SYCP1 constructs in COS-7 cells. The numbers correspond to the construct boundaries of the amino acid sequence. Scale bars, 20 μm. (**C**) SYCP1 truncations were investigated in MCF7 SYCP1 KO cells. FL, full length. Scale bars, 10 μm. (**D**) MCF7 SYCP1 KO cells were transfected with full length and truncated SYCP1 and tracked with Incucyte live imaging for 90 hours. Error bars represent the SEM.

SYCP1 is a 976–amino acid coiled-coil protein that mediates synapsis between homologous chromosomes during meiosis. This is achieved through the C-terminal binding to chromosomal DNA, with the N-terminal ends of juxtaposed SYCP1 molecules interacting head-to-head in a zipper-like manner midway between synapsing chromosomes (*11*, *24*). Hence, we wondered whether the pro-proliferative effect of SYCP1 is due to its DNA binding activity and/or its head-to-head self-assembly (Fig. 6A). To test this, we analyzed a C-terminal construct (amino acids 640 to 976) that is dimeric as it contains the end of the coiled-coil, includes the full DNA binding region, and was previously shown to bind to DNA *in vitro* (*24*). Upon overexpression, SYCP1 640 to 976 had localized to chromatin (Fig. 6, B and C) and stimulated growth in KO MCF7 cells at a level comparable to the full-length protein (Fig. 6D). Thus, we conclude that the pro-proliferative effect of SYCP1 is mediated solely by its DNA binding region and does not depend on self-assembly of its N termini.

The SYCP1 640 to 976 construct contains a dimeric coiled-coil (amino acids 640 to 783), followed by predicted unstructured sequence, which includes substantial basic charge at its C-terminal end that likely mediates, or contributes to, its DNA binding (Fig. 6A). Accordingly, we found that deletion of the last 22 amino acids of the protein (in construct 640 to 954) abrogated the stimulation of growth (Fig. 6D) while partially retaining nuclear localization (Fig. 6, B and C). Hence, DNA binding mediated by basic amino acids at the C-terminal tip of the protein is required for SYCP1’s pro-proliferative effect. We reasoned that the basic patches within SYCP1’s C terminus may have dual roles in DNA binding and nuclear localization (fig. S11). Hence, to separate these functions, we explored mutants of the C terminus in which basic patches remain intact. As part of a parallel study, we identify an internal deletion (Δ918–942) (Fig. 6A) that had a similar effect in abrogating growth stimulation while retaining nuclear localization (Fig. 6B). This deletion does not include overt DNA binding basic patches but instead removes divergent intervening sequence. Thus, it retains nuclear localization and putative DNA binding sites, suggesting that the wider architecture of SYCP1’s C terminus is important for its pro-proliferative effect.

**Fig. 7.**
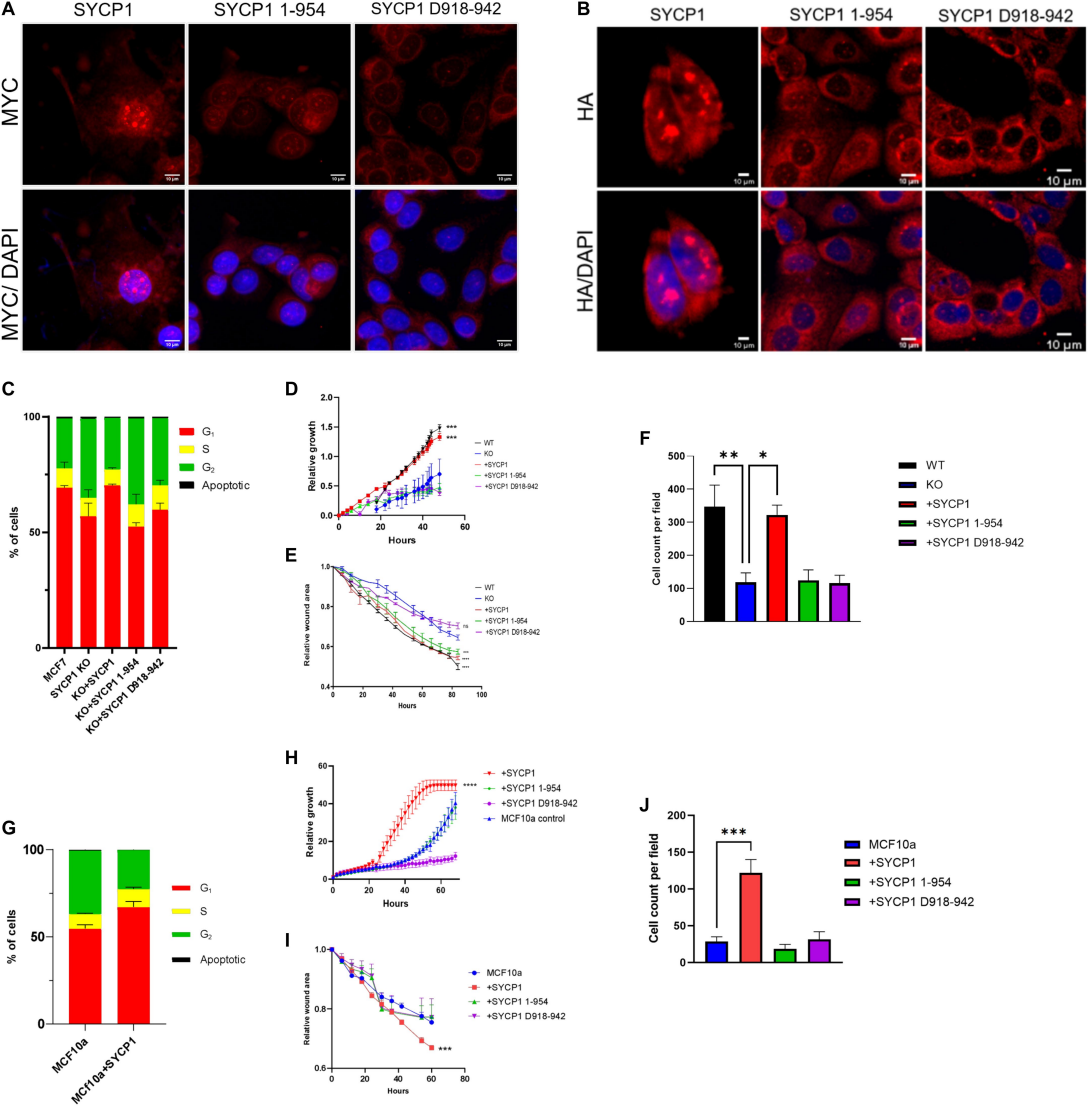
Nuclear localization of SYCP1 is critical for pro-proliferative and migratory function. (**A** and **B**) SYCP1 truncation 1 to 954 and deletion D918–942 were investigated in MCF7 cells after identification as possible NLSs. Nuclear localization was completely lost in D918–942, and a reduction was observed in SYCP1 1 to 954, for both the N-terminal MYC-tagged SYCP1 (A) and the C-terminal hemagglutinin (HA)–tagged SYCP1 (B). (**C**) Cell cycle function was investigated through the use of guava flow, and the shorter G_1_ observed in KO cells was rescued only by the nuclear localized full-length SYCP1. (**D** and **E**) MCF7 SYCP1 KO cells were transfected with full-length, 1 to 954, and D918–942 SYCP1 and tracked with Incucyte live imaging for 90 hours, and only the full length rescued proliferation (D) and wound closure rate (E). (**F**) Cells were seeded at equal density into Transwells in serum-starved media with complete media at the other side of the chamber. After 24 hours of incubation, migrated cells were counted, only full-length SYCP1 rescued KO cells to WT levels of migration. Untransfected MCF10a WT cells compared to MCF10A cells stably transfected with SYCP1 were subjected to cell cycle analysis (**G**). WT MCF10a cells and cells transfected with SYCP1 variants were seeded at equal density for 90 hours, and both proliferation (**H**) and wound closure (**I**) rate were determined. (**J**) WT MCF10a cells and cells transfected with SYCP1 variants were seeded at equal density into Transwells in serum-starved media with complete media at the other side of the chamber. After 24 hours, cells that migrated through the Transwell were counted, and only full-length SYCP1 increased the MCF10a cell migration rate. In all subfigures, error bars represent the SEM. Scale bars, 10 μm.

Having demonstrated these effects using the C-terminal 640 to 976 construct, we next assessed the same deletions within full-length SYCP1. We found that the SYCP1 1 to 954 truncation had decreased nuclear localization, whereas the del918–942 variant was largely unable to localize to the nucleus (Fig. 7, A and B). This is in keeping with our observation that 640 to 954 is only partially nuclear, indicating the presence of a nuclear localization signal (NLS) at the C-terminal end of SYCP1. In contrast, failure of full-length D918–942 to localize to the nucleus, despite this function being retained in 640 to 976, suggests that robust chromatin-associated structures are necessary to overcome the propensity of full-length SYCP1 to form cytoplasmic structures and retain nuclear localization. We performed rescue experiments in MCF7 *SYCP1* KO cells and found that, although full-length SYCP1 was able to rescue all cellular function to WT levels, variants with nuclear localization defects were not able to restore the cell cycle profile (Fig. 7C), proliferation (Fig. 7D), or migration (Fig. 7, E and F). The same relationship was observed when SYCP1 variants were introduced to MCF10a cells (Fig. 7, G to J).

### SYCP1 coimmunoprecipitates with promoter-specific DNA binding complexes that modulate TF activity

**Fig. 8.**
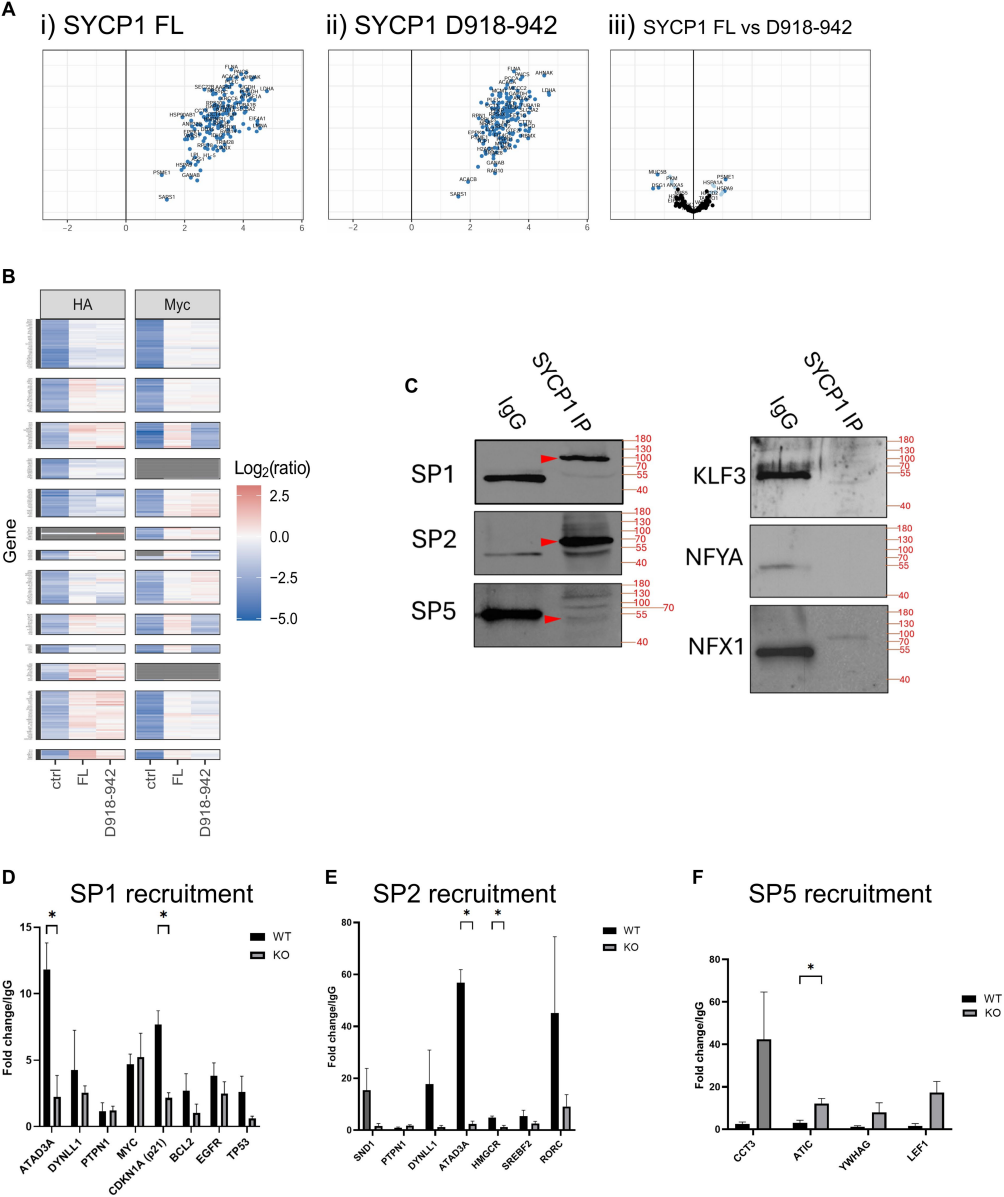
BioID2 interactome of WT SYCP1 versus D918–942. MCF7 cells with stable integration of BioID2-SYCP1 or BioID2-SYCP1 D918–942 were biotinylated for 24 hours. Pull-downs were sent for mass spectrometry TMT analysis. (**A**) Detected SYCP1 proximal proteins were compared to control in SYCP1 (i) and SYCP1 D918–942 (ii) as well as to each other (iii). (**B**) Thirteen clusters were detected on the basis of expression patterns between the control (ctrl), full-length, and truncated versions tagged at both the N and C termini. (**C**) Western blot of SP1, SP2, SP5, KLF3, NFYA, and NFX1 from SYCP1 immunoprecipitation (IP) in MCF7 cells. (**D**) SP1 ChIP-qPCR of SYCP1 target genes including shared SYCP1 and SP1 targets *ATAD3A, p21*, and *DYNLL1*. Data were normalized to IgG control and represent three biologically independent repeats; error bars show the SEM. (**E**) SP2 ChIP-qPCR of SYCP1 target genes including shared SYCP1 and SP2 targets *SIND1, DYNLL1*, and *ATAD3A*. Data were normalized to IgG control and represent three biologically independent repeats; error bars show the SEM. (**F**) SP5 ChIP-qPCR of SYCP1 target genes including shared SYCP1 and SP5 targets *ATIC* and *YWHAG*. Data were normalized to IgG control and represent three biologically independent repeats; error bars show the SEM.

To elucidate the molecular mechanisms by which SYCP1 regulates gene expression in cancer cells, we used proximity-dependent biotin labeling (BioID2) to define the SYCP1 interactome. Mass spectrometry analysis revealed that SYCP1 associates with chromatin-bound proteins, including core histones and chromatin-modifying enzymes. Notably, SYCP1 was enriched in complexes involved in promoter-specific DNA binding, a pattern that was corroborated by SYCP1 CUT&Tag profiling, which revealed colocalization with gene promoters and enriched signal over single-stranded and oxidized DNA regions (Fig. 8A, i and ii). SYCP1 was also found in proximity to histone methyltransferases and DNA endonucleases, suggesting a broader role in chromatin state modulation.

To probe the functional relevance of SYCP1’s C-terminal region, we compared the interactomes of WT SYCP1 and a truncated mutant lacking amino acids 918 to 942 (Δ918–942), which fails to rescue SYCP1-dependent phenotypes in cancer cells (Fig. 7). BioID2-tagged SYCP1 constructs (WT and Δ918–942) and empty vector controls were stably expressed in MCF7 cells, biotinylated for 24 hours, followed by streptavidin enrichment and tandem mass tag (TMT)–based quantitative proteomics (Fig. 8A, i to iii). Comparative analysis revealed distinct interaction profiles between WT and mutant SYCP1, with 13 discrete protein clusters showing differential proximity (Fig. 8B). The Δ918–942 variant lost binding to nuclear proteins and, critically, failed to interact with mitotic cell cycle regulators and components of adhesion complexes including cadherin-associated proteins, focal adhesion machinery, and cell-cell junctional proteins.

Integrating SYCP1’s BioID2 interactome with CUT&Tag co-occupancy data, we prioritized a set of TFs that are likely to cofunction with SYCP1 at chromatin. These included SP1, SP2, SP5, KLF3, NFYA, and NFX1. Coimmunoprecipitation experiments in MCF7 cells demonstrated that SYCP1 pulls down SP1, SP2, and SP5 but not KLF3, NFYA, or NFX1 (Fig. 8C).

To test whether SYCP1 regulates the chromatin association of SP family TFs, we examined SP1, SP2, and SP5 occupancy at shared target genes upon SYCP1 loss (Fig. 8, D to F). Chromatin immunoprecipitation and qPCR (ChIP-qPCR) revealed that SYCP1 depletion impaired SP1 and SP2 binding at cotargeted promoters (Fig. 8, D and E), whereas SP5 occupancy was conversely increased (Fig. 8F), suggesting a competitive or compensatory regulatory relationship. These findings indicate that SYCP1 modulates promoter accessibility and transcriptional output through specific interaction with and regulation of SP family TFs, positioning SYCP1 as an integral component of oncogenic gene regulatory circuits in cancer cells.

### SYCP1 localizes to sites of DNA damage and modulates chemotherapy response

In its canonical meiotic role, SYCP1 is a core component of the SC, which bridges homologous chromosomes and facilitates the repair of programmed DSBs during meiosis (*11*). Our findings in cancer models revealed that SYCP1 is aberrantly expressed in the nucleus, binds chromatin, and regulates transcription of DDR and repair genes (Figs. 2 to 4). Given this context, we hypothesized that SYCP1 might be functionally responsive to genotoxic stress and directly localize to DNA damage sites.

**Fig. 9.**
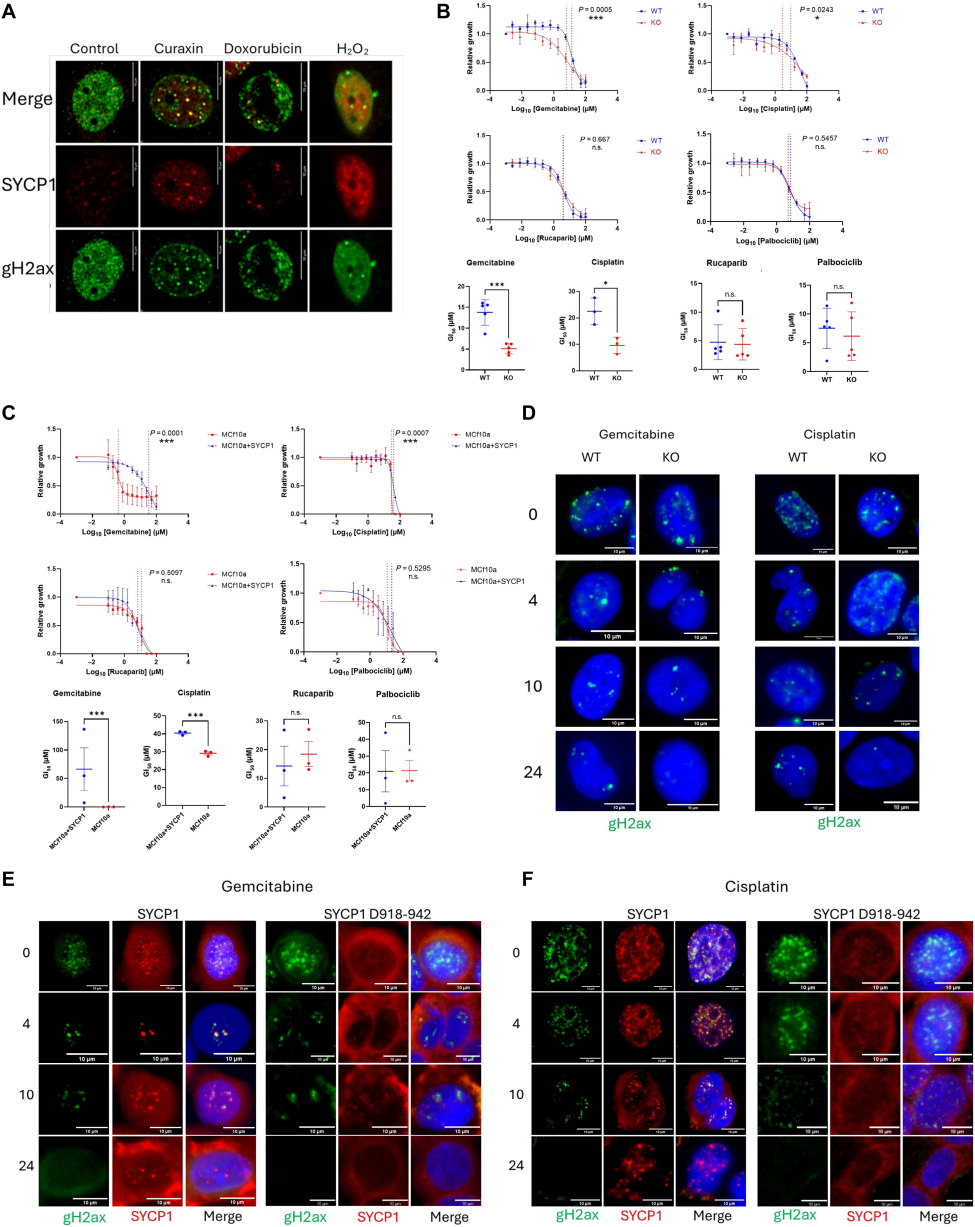
SYCP1 promotes resistance to chemotherapy in breast cancer cells. (**A**) Colocalization of DNA damage marker H2AX and SYCP1 in a SYCP1 Dox-inducible MCF7 cell line. Scale bars, 10 μm. (**B**) Median growth inhibition (GI_50_) values of WT and SYCP1 KO MCF7 cells in response to gemcitabine, cisplatin, rucaparib, and palbociclib. Error bars represent the SEM. (**C**) GI_50_ values of MCF10a cells and MCF10a transfected with SYCP1 in response to gemcitabine, cisplatin, rucaparib and palbociclib. Error bars represent the SEM. (**D**) Colocalization of DNA damage marker gH2ax and SYCP1 in MCF7 WT or KO cells, at 0 hours (damage induction) and after 4, 10, or 24 hours of recovery, in response to gemcitabine or cisplatin. (**E**) Colocalization of gH2ax and SYCP1 or SYCP1 D918–942 at 0, 4, 10, or 24 hours after DNA damage induction using gemcitabine. Scale bars, 10 μm. (**F**) Colocalization of gH2ax and SYCP1 or SYCP1 D918–942 at 0, 4, 10, or 24 hours after DNA damage induction using cisplatin. Scale bars, 10 μm. n.s., not significant.

Upon exposure to DNA-damaging agents, we observed robust colocalization of SYCP1 with γH2AX foci, a marker of DSBs (Fig. 9A), indicating that SYCP1 is actively recruited to DNA damage sites. Given that patients with high SYCP1 expression exhibit significantly shorter overall and relapse-free survival across multiple cancer types (fig. S1), we investigated whether SYCP1 contributes to chemoresistance by facilitating DNA repair.

We tested this hypothesis using the chemotherapeutics gemcitabine and cisplatin, a combination frequently used in neoadjuvant therapy for nonoperable breast cancer (*23*). SYCP1 KO sensitized MCF7 breast cancer cells to both drugs (Fig. 9B) while having no impact on the response to rucaparib (a PARP1 inhibitor) or palbociclib (a CDK4/6 inhibitor). Similarly, no impact was observed in response to MYCi361, doxorubicin, SN-38, or radiation (fig. S8). A similar drug response profile was observed in nontransformed MCF10A cells ectopically expressing SYCP1, compared to untransfected controls (Fig. 9C), suggesting that SYCP1 enhances resistance to genotoxic stress by modulating early stages of the DNA repair cascade.

To further explore SYCP1’s function in DNA repair kinetics, we examined the formation and resolution of γH2AX foci following chemotherapeutic insult (Fig. 9D). In WT cells, gemcitabine and cisplatin treatment induced pronounced γH2AX foci that persisted for up to 24 hours (fig. S9). In contrast, SYCP1-deficient cells formed fewer foci with more rapid resolution, suggesting that SYCP1 may contribute to foci formation and stabilization of the damage response.

We next examined whether SYCP1 is physically retained at DNA damage sites during repair. Using immunofluorescence, we analyzed SYCP1 and γH2AX localization over a 24-hour recovery period in cells expressing either full-length SYCP1 or the Δ918–942 mutant. Full-length SYCP1 colocalized with γH2AX immediately following DNA damage induction by gemcitabine and cisplatin (Fig. 9E). In contrast, the Δ918–942 variant—defective in nuclear localization—exhibited minimal recruitment to γH2AX-marked foci (Fig. 9E).

Temporal analysis revealed sustained colocalization of full-length SYCP1 with γH2AX during recovery, particularly after cisplatin exposure (Fig. 9F). SYCP1 foci persisted even at 24 hours posttreatment, at which point γH2AX foci were largely resolved. This suggests that SYCP1 remains associated with formerly damaged loci, potentially stabilizing repaired chromatin or contributing to late-stage events in homologous recombination.

Together, these data position SYCP1 as a regulator of the DDR in cancer cells, facilitating both recruitment to DNA lesions and influencing repair kinetics—mechanistic features that may underlie its association with poor clinical outcomes and chemoresistance.

### SYCP1 in patients with breast cancer correlates with poor prognosis

**Fig. 10.**
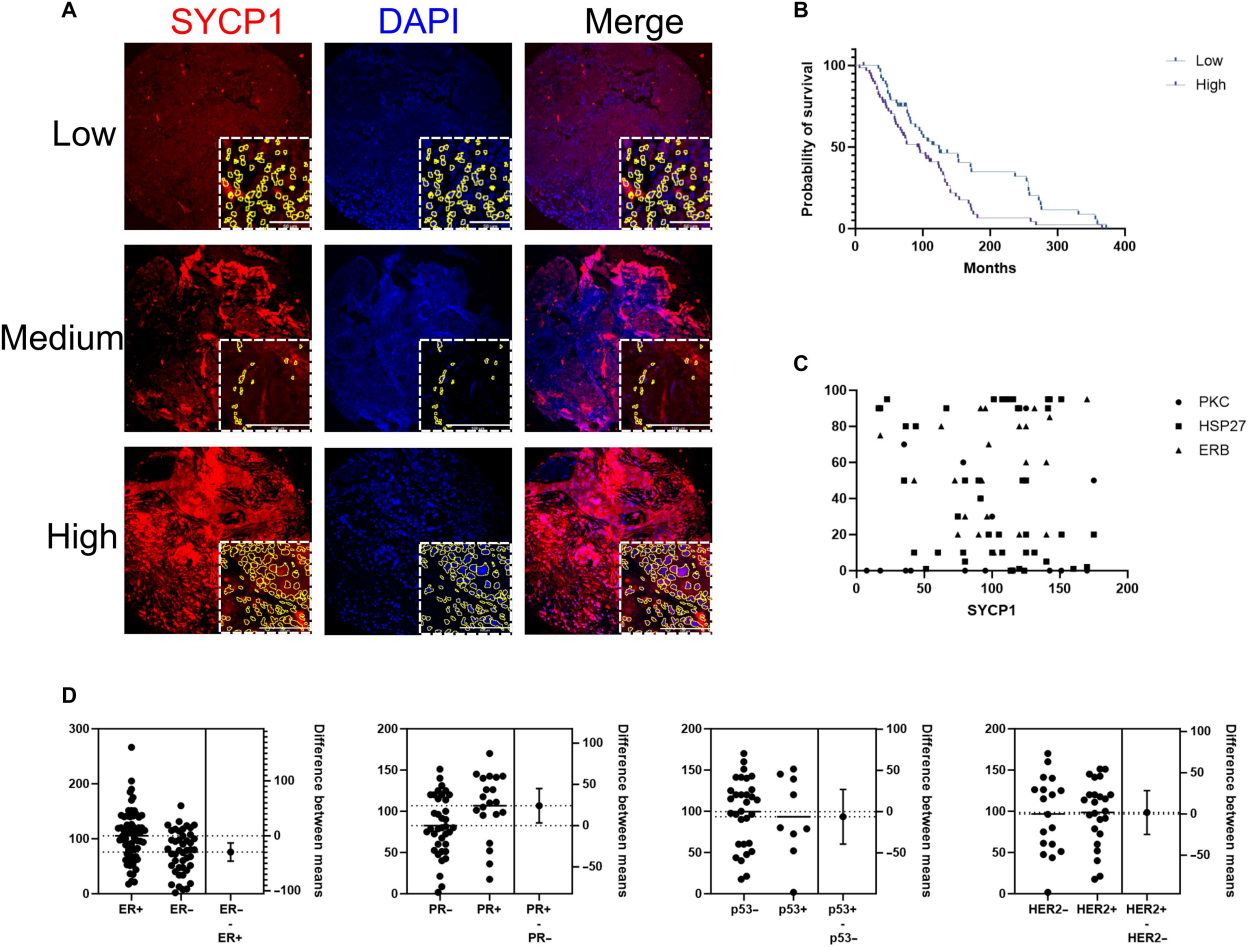
SYCP1 hinders DNA damage repair in patients with breast cancer. (**A**) TMAs from patients with breast cancer were immunolabeled for SYCP1; SYCP1 levels vary between patients. Yellow demarcation indicates nuclei outlines. (**B**) Kaplan-Meier survival analysis in patient’s subgroups with high and low SYCP1 protein. Gehan-Breslow-Wilcoxon test, *P* = 0.0191. Median survival: SYCP1 low, 124.0; SYCP1 high 95.00; ratio (and its reciprocal): 1.305, 0.7661; 95% confidence interval of ratio: 0.8721 to 1.954; 0.5119 to 1.147, *n* = 114. (**C**) No correlation between SYCP1 and PKC, HSP27, or ERB. (**D**) Both patients with positive ER and positive PR had higher histoscores. Histoscores remained were not significantly different based on the P53 or HER2 patient status.

To investigate the clinical relevance of SYCP1 reexpression, we analyzed a large cohort of patients with breast cancer (*n* = 137), focusing on protein-level expression due to the previously noted discordance between SYCP1 mRNA and protein abundance (Fig. 1). Immunohistochemical analysis revealed that SYCP1 protein was aberrantly expressed in the vast majority of patient samples (Fig. 10A), as well as in patients with kidney liver and sarcoma cancer (fig. S10). Notably, SYCP1 expression did not correlate with the expression of unrelated stress markers such as HSP27 or PKC (Fig. 10C), indicating specificity in its association with DNA repair machinery.

We next examined whether SYCP1 expression was associated with clinical features in breast cancer. Patients with positive estrogen receptor (ER+) exhibited significantly higher SYCP1 protein levels compared to patients with negative ER (ER−) (Fig. 10D), a trend also observed for progesterone receptor (PR) status (Fig. 10D). However, SYCP1 levels were not significantly associated with *TP53* mutational status or homologous recombination (HR) proficiency (Fig. 10D), suggesting that SYCP1 expression is not merely a surrogate marker of global genomic instability.

Critically, high SYCP1 expression was significantly associated with reduced overall survival in patients with breast cancer (Fig. 10B), reinforcing its potential as a clinically relevant marker of poor prognosis. These findings suggest that aberrant expression of SYCP1 in cancer may impair DNA repair fidelity, promoting genome instability and therapeutic resistance.

## DISCUSSION

**Fig. 11.**
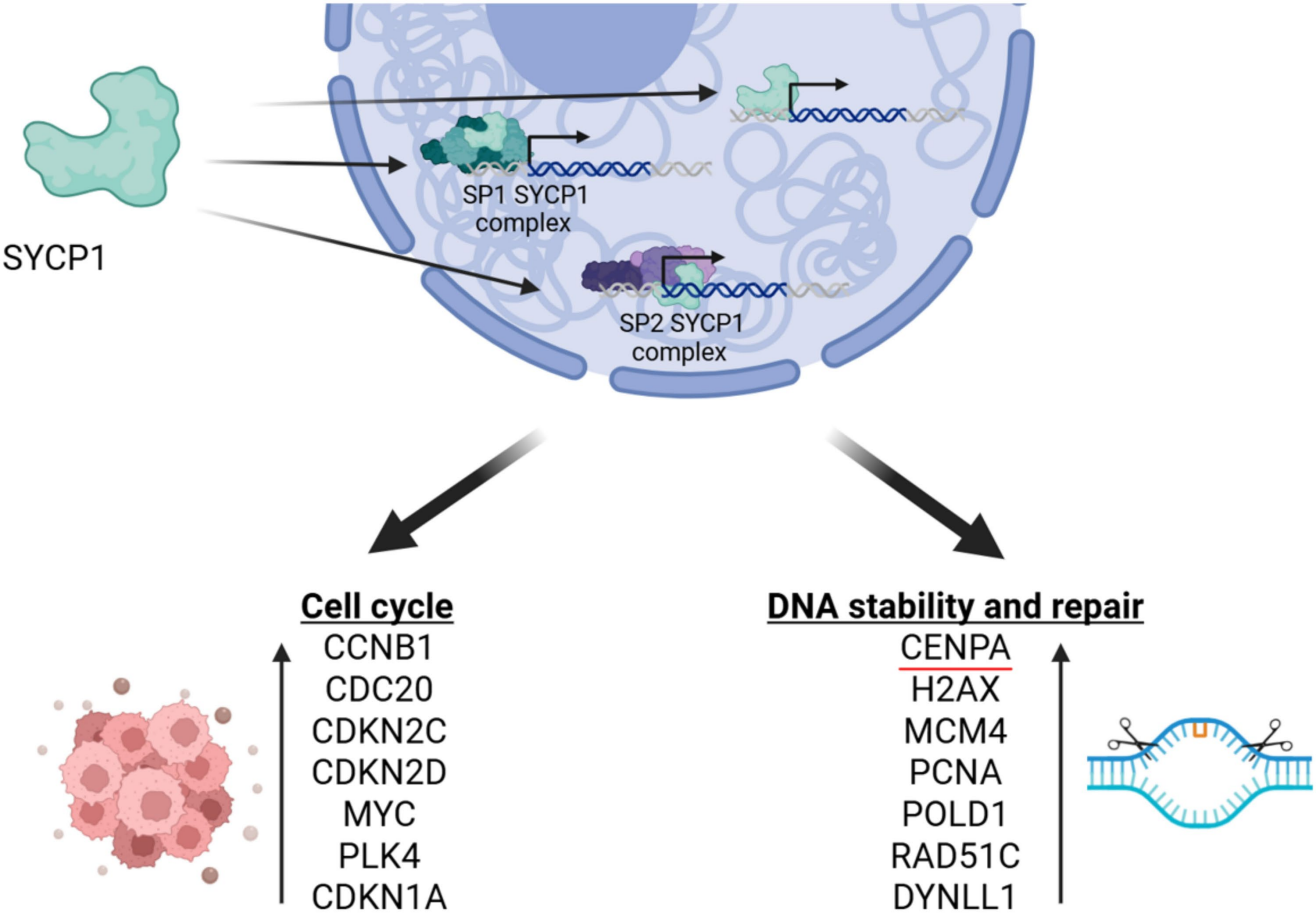
Proposed model of SYCP1 function in cancer cells. In cancer cells, the normally meiotic protein SYCP1 is aberrantly expressed and translocates to the nucleus, where it adopts a noncanonical regulatory role. Once nuclear, SYCP1 associates with the promoters of a distinct subset of genes, including cell cycle– and proliferation-related targets, via interactions with the TFs SP1 and SP2. This complex promotes transcriptional activation, leading to increased expression of oncogenic programs. SYCP1-driven gene expression changes are associated with enhanced proliferative capacity, resistance to DNA damage, and disruption of normal chromatin architecture. Our model suggests that SYCP1 acts as a chromatin-bound transcriptional coregulator in cancer, repurposed from its canonical structural role in meiosis. Together, our data show that SYCP1 expression in cancer cells is associated with a transcriptional program enriched for cell cycle and stress response genes, which is consistent with the observed increases in proliferation and altered response to DNA-damaging agents. Although our results support a role for nuclear/chromatin-associated SYCP1 in rewiring the gene expression, the precise mechanistic link between this transcriptional program and the migration/invasion phenotype remains less well defined and should be addressed in future work. Created in BioRender. McClurg, U. (2026) https://BioRender.com/hw4a7qj.

Our study uncovers an unexpected oncogenic role for the meiotic protein SYCP1 in somatic cancer cells, where we propose that SYCP1 regulates transcriptional programs, influences DNA damage repair, and promotes tumor progression (Fig. 11). Classically defined as a structural component of the SC essential for meiotic homologous recombination (*10*), SYCP1 is aberrantly reexpressed in cancer and repurposed for noncanonical functions in genome maintenance. These findings underscore a broader paradigm in tumor biology, cancer cells exploit developmental pathways, co-opting germline proteins to overcome genomic instability and therapeutic pressure.

### Moonlighting functions of SYCP1 beyond structural support

In meiosis, SYCP1 facilitates chromosome synapsis by bridging homologous chromosomes (*22*). In contrast, in cancer cells, we show that SYCP1 localizes to chromatin, where it regulates gene expression programs associated with cell cycle control and DNA repair. Mechanistically, SYCP1 modulates chromatin recruitment of SP family TFs, particularly SP1 and SP2, which are key regulators of TATA-less housekeeping genes. SP1 is a well-established oncogenic TF that promotes proliferation in various malignancies, including breast cancer (*24*), and correlates with poor clinical outcomes (*25*). SP2 similarly contributes to tumor formation (*26*). Conversely, SP5 has more limited expression and has been linked to pro-apoptotic signaling, including as a WNT pathway target (*27*). Our data suggest that SYCP1 might promote tumorigenesis by enhancing SP1 and SP2 recruitment to chromatin while suppressing SP5 activity, which would collectively drive cell cycle progression and migration. However, it remains unknown whether SYCP1 binds directly to SP1 and SP2, whether they are part of a wider complex network or whether SYCP1 alters local chromatin structure in a manner that facilitates SP1 and SP2 recruitment.

Our proximity labeling and CUT&Tag experiments reveal that SYCP1’s interactome is context dependent, shifting from structural synapsis-associated partners in meiosis to chromatin regulators and TFs in cancer. This functional plasticity mirrors findings for other meiotic proteins aberrantly reexpressed in tumors. For instance, SPO11 and HORMAD1—essential for meiotic recombination—have been shown to disrupt DNA repair when expressed in somatic cells (*4*, *22*, *28*).

### SYCP1 and DNA damage repair: A double-edged sword?

Our data show that SYCP1 regulates the transcription of key DNA repair genes, including BARD1, RAD51C, and H2AX. Loss of SYCP1 sensitizes cancer cells to DNA-damaging agents such as cisplatin and gemcitabine, indicating a role in promoting repair. However, rather than supporting high-fidelity repair, SYCP1 may facilitate a noncanonical, potentially error-prone pathway that enables cancer cell survival. This is consistent with the idea that tumors often rely on alternative repair strategies to maintain viability under genotoxic stress.

A key finding is the persistent colocalization of SYCP1 with γH2AX-marked foci following DNA damage. These foci remain even after γH2AX has largely resolved, suggesting that SYCP1 may participate in the late stages of homologous recombination or contribute to stabilization of repaired chromatin. The persistence of SYCP1 at these sites raises important questions: Does SYCP1 promote accurate repair, or does it interfere with proper resolution? Given the association between defective repair, chromosomal aberrations, and aggressive tumor behavior, further mechanistic studies are warranted to elucidate whether SYCP1 contributes to mutagenic repair outcomes that drive clonal evolution and resistance.

## MATERIALS AND METHODS

### Cell culture and transfections

MCF7 breast cancer cells, MCF10 noncancerous breast epithelial cells, U2OS osteosarcoma, LNCaP, CWR22Rv1 and PC3 prostate cancer cells, COS-7 epithelial cells, and PC9 lung cancer cells were obtained from the American Type Culture Collection (Manassas, USA). Mel270 uveal melanoma cells were a kind gift from S. Coupland (Liverpool University). PEO1 ovarian cancer cells were a kind gift from the NICR Cell Bank (Newcastle University). WM164 and WM1361 melanoma cells were a kind gift from P. Lovat (Newcastle University). PC9 cells were a kind gift from P. Eyers (Liverpool University). A375, SNU16, and AGS stomach cancer cells, as well as SKMEL1 melanoma cells, were from the WWT Lab collection. THP1 monocytic leukemia, NALM6 B cell precursor leukemia, HL60 promyelocytic leukemia, Caco2 and LoVo colorectal carcinoma, JIMT1 breast cancer, Hep3B liver cancer, and U937 histiocytic lymphoma cells were a kind gift from L. Pulaski (Institute of Medical Biology). MCF7 Fucci cells were a kind gift from R. Mort (Lancaster University). Cell lines were maintained in Dulbecco’s modified Eagle’s medium (DMEM) supplemented with 2 mM l-glutamine (Invitrogen) and 10% (v/v) fetal calf serum (FCS) at 37°C in 5% CO_2_. Cell lines were never maintained for more than 30 passages or 2 months of continuous culturing. Cell lines were tested for mycoplasma on a trimonthly basis. Proliferation was measured by live cell imaging with the Incucyte system every 6 hours for 156 hours posttreatment. Transfections were performed using Lipofectamine 2000 reagent (Invitrogen) following the manufacturer’s instructions.

### Generation of a Dox-inducible SYCP1-expressing MCF7 line

SYCP1 was overexpressed in MCF7 cells using the pCW-SYCP1_3xTy1 vector. The vector was transfected into MCF7 cells, and a stable clone was established. SYCP1 expression was induced with Dox (5 μg/ml).

### siRNA gene silencing and gene expression analysis

The *SYCP1* targeting small interfering RNA (siRNA) sequences were (A) GACUCUGAUUUGGAGAAUUCA[dTdT], (B) GAGUUUCCAUUUGCAAAGACU[dTdT], and (C) GUCACUAUCAGGAAGGACU[dTdT] (Sigma-Aldrich). Cells were reverse transfected with siRNA using RNAiMax (Invitrogen) according to manufacturer’s instructions and incubated in culture media for 96 hours before cell lysis and analysis. For real-time qPCR, extraction of RNA was carried out using TRIzol (Invitrogen, catalog no. 15596026) following the manufacturer’s instructions. One microgram of RNA was incubated at 65°C for 5 min, followed by incubation at 37°C for 2 min. cDNA was synthesized using the M-MLV Reverse Transcriptase (Promega, catalog no. M1701). For qPCR, we used the SYBR Green PCR Master Mix (Invitrogen, catalog no. 4309155) and StepOne systems (Applied Biosciences, catalog no. 4376357), and each sample was normalized to the *HPRT1* housekeeping gene. Three biological replicates were carried out for each reaction. Fold changes were calculated using the 2^−ΔΔCT^ relative quantification method. Data were tested for parametric distribution. Parametric data were analyzed using appropriate *t* tests or analysis of variance (ANOVA) with Bonferroni’s comparison test for multiple group comparisons. Nonparametric data were analyzed using Wilcoxon signed-rank test. By convention, ***P < 0.001, ** < 0.01 and * < 0.05.

### Genotyping CRISPR KO clones

SCYP1 KO was generated through CRISPR-Cas9–mediated genome editing in MCF7 cells and was carried out by Synthego, using a guide RNA (gRNA) targeting exon 2 (5′-AAGCAGCAGTCAGGTGTCTG-3′). Single clones were genotyped using the Zero Blunt TOPO PCR Cloning Kit (Invitrogen, catalog no. 450245) according to the manufacturer’s instructions. Upon bacterial transformation, colonies were selected for plasmid isolation using the ZymoPURE Plasmid Miniprep Kit (Zymo Research, catalog no. D4214) and subsequently subjected to Sanger sequencing to identify editing events.

### Immunofluorescence

For Immunofluorescence, cells were fixed for 10 min in ice-cold methanol followed by blocking in 1% bovine serum albumin (BSA), staining overnight at 4°C in primary antibodies and then for 1 hour at room temperature with goat anti-rabbit Alexa Fluor 594 (A32740) or Alexa Fluor 647 (A-21245) and rabbit anti-mouse Alexa Fluor 488 (A-11059) secondary antibodies (Life Technologies) and 4′,6-diamidino-2-phenylindole (DAPI). Mounting was performed in ProLong Antifade Gold Reagent (Life Technologies, P36961) or VECTASHIELD Antifade Medium (H-1000-10), and imaging was performed using an LSM 880 confocal microscope and processed with the Zen 2.6 Blue Edition software.

### Immunohistofluorescence

Tissue microarrays (TMAs) containing 0.6-mm cores of breast cancer and control tissues including sarcoma, liver, colon, and kidney cancer from retrospective cohorts were used. Our cohort included patients treated within the Merseyside Hospital Trust in the 1990s and 2000s. Antigens were retrieved by microwaving the slides in 10 mM citrate (pH 6.0) for 15 min followed by immunofluorescence. Samples were scored blind using the histoscore methodology (*25*). Briefly, percentage and intensity of staining for positive cells were estimated (0, 1, 2, 3) using the following equation: *H*-score = (% of cells with low-level positivity) + 2 × (% of cells with medium-level positivity) + 3 × (% of cells with high level positivity). For survival analysis, high marker levels were defined as a value in the third and fourth upper quarter of the population. Tissues were imaged at the Liverpool University Centre for Cell Imaging (CCI).

### Chromatin fractionation

Cells were washed with phosphate-buffered saline (PBS) and trypsinized and pelleted. The cell pellets were washed with ice-cold PBS and lysed with CSK buffer [10 mM Pipes (pH 7.0), 200 mM NaCl, 300 mM sucrose, 3 mM MgCl_2_, 1 mM dithiothreitol (DTT), 1 mM aprotinin, 1 mM pepstatin, 1 mM leupeptin, and 2 mM phenylmethylsulfonyl fluoride (PMSF)] containing 0.5% (v/v) Triton X-100 and incubated on ice for 10 min. The lysate was then spun down at 1500*g* for 5 min at 4°C. The supernatant was spun down at maximum speed for 30 min at 4°C. The pellet (chromatin fraction) was washed twice with CSK buffer containing 0.1% (v/v) Triton X-100. The pellet was resuspended in buffer C [20 mM Tris (pH 7.9), 1.5 mM MgCl_2_, 420 mM KCl, 1 mM DTT, 1 mM aprotinin, 1 mM pepstatin, 1 mM leupeptin, and 2 mM PMSF] and incubated in a cold room while rotating for 90 min, followed by five cycles of sonication on high setting. The chromatin fraction was then spun down at maximum speed for 30 min at 4°C. The supernatant was collected for downstream Western blotting.

### Colony formation assay

Cells were seeded in a 6-well plate at a density of 1000 cells per well, left for 21 days, and then washed with PBS and stained with crystal violet overnight.

### Transwell migration assay

Cells were seeded in the Transwell inserts in serum-free media and incubated for 24 hours. After incubation, the cells were washed once with PBS and fixed with ice-cold 75% (v/v) ethanol for 10 min, followed by additional washing with PBS. Crystal violet was added for overnight staining. Transwell inserts were then imaged using a microscope, and representative images were selected and processed using ImageJ.

### Anchorage-independent growth

Cells were plated into media containing 0.56% methylcellulose in a 24-well plate and incubated at 37°C in 5% CO_2_ for 14 days followed by imaging with Incucyte and automated colony counting.

### MCF7-H2B-Fucci2a live cell cycle analysis

The PiggyBac compatible pB-H2B-Cerulean-Fucci2a plasmid (*26*) was a kind gift from R. Mort (Lancaster University). pB-H2B-Cerulean-Fucci2a and pbHybase were transfected into MCF7 cells using Genejuice (70967-3, Merck). Stable transfectants were selected with puromycin, and individual clones were grown.

For live cell imaging, MCF7-H2B-Fucci2a cells were seeded onto glass-bottom 24-well plates (P24-1.5P, Cellvis) and reverse transfected (Lipofectamine RNAiMax) with 10 nM control or siSYCP1_B siRNAs. Twenty-four hours after transfection, live cell imaging was performed on a Zeiss LSM 880 confocal microscope equipped with Focus Controller 2, with cells maintained at 37°C and 5% CO_2_ using the TempModule S1 (800-450000, Pecon) and CO_2_ Module S1 (810-450001, Pecon). Images were acquired every 15 min for 65 hours using a Plan-Apochromat 20x/0.8 M27 objective at Lancaster University. To determine cell cycle parameters, the daughters of a mitosis were tracked in FIJI, either until they completed two full cell cycles or for the duration of the time-lapse.

### RNA extraction and RNA-seq library preparation

At least 5 × 10^5^ cells were lysed using TRIzol reagent, and RNA was extracted with the Direct-zol RNA MiniPrep Kit (Zymo Research, catalog no. R2050) according to the manufacturer’s protocol. One microgram of RNA was used as input for library preparation. Ribosomal RNA (rRNA) depletion was performed using the NEBNext rRNA Depletion Kit v2 (NEB, catalog no. E7405), followed by cDNA synthesis, end prep, and library amplification using the NEBNext Ultra II Directional RNA Library Prep Kit for 56 Illumina (NEB, catalog no. E7760). The average fragment length was determined using the D1000 ScreenTape (Agilent, catalog no. 5067-5582) with D1000 reagents (Agilent, catalog no. 5067-5583), and sample concentration was measured with the Qubit 3.0 Fluorometer (Life Technologies). Next-generation sequencing was performed by Macrogen (Singapore).

### RNA-seq data analysis

Trim Galore (v0.4.2_dev; https://bioinformatics.babraham.ac.uk/projects/trim_galore/) was used to trim paired-end raw sequencing reads. The trimmed reads were then mapped to the human hg19 reference genome (hg19/GRCh37) obtained from iGenomes (https://sapac.support.illumina.com/sequencing/sequencing_software/igenome.html) using the RSEM pipeline (v1.1.11) (*27*). Differential expression analyses were performed using DESeq2 (*28*). Differentially expressed genes (DEGs) were identified on the basis of a 1.5-fold change in expression and an adjusted *P* value of less than 0.05. Pathway analysis was conducted using the Metascape package (https://metascape.org/gp/index.html#/main/step1) and gene set enrichment analysis (GSEA) software (https://gsea-msigdb.org/gsea/index.jsp) using the Molecular Signatures Database (MSigDB). Pathways were considered significantly enriched if the false discovery rate (FDR)–corrected *P* value was ≤0.05. Volcano plots were generated using packages ggplot2 and EnhancedVolcano in the R software (v4.0.5).

### CUT&Tag assay

A total of 1 × 10^5^ of cells were resuspended in a wash buffer [20 mM Hepes-KOH (pH 7.5), 150 mM NaCl, and 0.5 mM spermidine]. Concanavalin A beads were prepared by washing twice with binding buffer [20 mM Hepes-KOH (pH 7.5), 10 mM KCl, 10 mM CaCl_2_, and 1 mM MnCl_2_]. Ten microliters of concanavalin A beads (Bangs Lab, catalog no. BP531) was added to each reaction and incubated with rotation at room temperature for 15 min. The cell-bead complex was resuspended in 50 μl of antibody buffer [20 mM Hepes-KOH )pH 7.5), 150 mM NaCl, 2 mM EDTA, 0.5 mM spermidine, 0.05% (w/v) digitonin, and 0.1% (w/v) BSA]. One microgram of anti-Ty1 primary antibody (Thermo Fisher Scientific, catalog no. MA5-23513) was added to each condition. The reactions were incubated at 4°C overnight with gentle rocking. After removing the supernatant containing the primary antibody using a magnet, the secondary antibody was added in dig-wash buffer [20 mM Hepes-KOH (pH 7.5), 150 mM NaCl, 0.5 mM spermidine, and 0.05% (w/v) digitonin] and incubated at room temperature for 1 hour with rotation. The complex was then washed twice with dig-wash buffer and resuspended in Dig-300 wash buffer [20 mM Hepes-KOH (pH 7.5), 300 mM NaCl, 0.5 mM spermidine, and 0.01% (w/v) digitonin]. One microliter of in-house pA-Tn5 transposase was added and incubated at room temperature with constant rotation for 1 hour. The complex was washed twice with Dig-300 wash buffer and resuspended in 300 μl of Dig-300 wash buffer with 10 mM MgCl_2_ and incubated at 37°C for 1 hour for tagmentation. The reaction was then quenched by adding 10 μl of 0.5 M EDTA, 3 μl of 10% (w/v) SDS, and 2.5 μl of proteinase K (20 mg/ml) and incubated at 50°C for 1 hour. DNA was purified with Serapure beads and eluted in 0.1x TE buffer. Libraries were prepared by tagging each sample with a unique pair of indexes provided in the NEBNext Multiplex Oligos for Illumina (NEB, catalog no. E7311AVIAL) following PCR. The libraries were purified with Serapure beads. The average fragment length was measured by the D1000 ScreenTape (Agilent, catalog no. 5067-5582) with D1000 reagents (Agilent, catalog no. 5067-5583). Next-generation sequencing was carried out by Macrogen (Singapore).

### CUT&Tag sequencing analysis

Each sample was sequenced with at least 20 million paired-end reads (150x150). Adapter, and quality trimming of the reads were performed using Trim Galore (https://github.com/FelixKrueger/TrimGalore) with the parameters --paired --nextera. The reads were aligned to the human genome hg19 using Bowtie2 (version 2.4.5) with the parameters --end-to-end --very-sensitive --no-mixed --no-discordant --phred33 -I 10 -X 700 (*22*). The Bowtie2 index was obtained from the Illumina iGenome website (https://sapac.support.illumina.com/sequencing/sequencing_software/igenome.html). Unmapped reads were removed using samtools view (https://htslib.org/doc/samtools.html) with the parameters -bS -F 0x04. Bigwig files and metagene plot were generated using deepTools (*29*). Comparisons between bed files (e.g., common overlapping and unique) were performed using bedtools (v2.30.0) (*30*). For peak calling, macs2 was used using the parameters BAMPE --keep-dup all -g hs -q 0.01 and immunoglobulin G (IgG) as a control (*31*). Pie charts were generated using ChIPseeker (*32*). Motif enrichment analysis was performed using HOMER (*33*) and BAMM (*34*).

### Public data analyses

Publicly available ATAC-seq data in MCF7 cells were obtained from Robey *et al.* (*23*). Raw data were downloaded from Gene Expression Omnibus (GEO accession: GSE217128). Trim_galore (https://github.com/FelixKrueger/TrimGalore) with the default options was used for reads filtering based on read quality and adapter sequence removal. The obtained fastq files were further aligned to the human reference genome (hg19) using STAR_2_7_10a (*35*) with the following parameters: “-alignIntronMax 1,” “-outFilterMismatchNoverLmax 0.09,” “-alignMatesGapMax 2000,” “-outFilterMultimapNmax 1,” and “-alignEndsType EndToEnd“; the rest of the options were set to the default. Duplicated reads were removed from the bam files using MarkDuplicates from the PICARD suite (http://broadinstitute.github.io/picard/). Repair of transposase-induced nick was done using the alignmentSieve function from deepTools (*29*). Significant ATAC-seq open chromatin regions were detected using the functions of macs2 software (*31*): (i) macs2 randsample to convert the bam to bed format and (ii) macs2 callpeak to identify significant ATAC-seq regions with the parameters: -f BEDPE --nomodel --shift −37 --extsize 73 -B --keep-dup all -g hs -q 0.01.

*SYCP1* mRNA expression across selected cancer subtypes was assessed using publicly available TCGA data accessed via cBioPortal. A TCGA pan-cancer dataset was selected, and the sample set was restricted to cases with available mRNA expression data. *SYCP1* expression was queried using mRNA expression *z*-scores (log RNA-Seq V2 RSEM), calculated relative to all samples. A *z*-score threshold of ±1 was applied to identify samples with aberrant *SYCP1* expression.

### BioID2 pull-downs

SYCP1 full length, variant 1–954, or deletion D918–942 were cloned into BioiD2-MYC or BioID2-HA plasmids. MCF7 cells were transfected using Lipofectamine 2000 according to the manufacturer’s instructions and selected with G418 (Geneticin; 400 μg/ml) for 2 weeks. MCF7 cell lines stably expressing BioID2 constructs were seeded in four 10-cm dishes at 3 × 10^6^ per dish, for each repeat. Biotin was added at 50 μM for 24 hours before cells were lysed and sonicated in the lysis buffer [50 mM tris-HCl (pH 7.4), 8 M urea, 1x protease inhibitor, and 1 mM DTT] and 20% Triton X-100. Lysates were centrifuged at 4°C and 13,000*g* for 10 min, and the supernatant was incubated with 100 μl of streptavidin-conjugated beaded agarose (Thermo Fisher Scientific) overnight on a roller at 4°C. Beads were washed three times in a wash buffer [50 mM tris-HCl (pH 7.4) and 8 M urea] and then spun for 2 min at 1000*g*. The supernatant was removed, and the pellet was resuspended in 30 μl of 25 mM biotin in 50 mM ammonium bicarbonate and heated at 95°C for 5 min before centrifugation for 2 min at 1000*g*. Without removal of the supernatant, this process was repeated. Eighty microliters of Laemmli buffer with b-mercaptoethanol was added, and the solution heated for 5 min at 95°C. The solution was frozen at −80°C for 30 min before a final centrifugation. The resultant supernatant was removed and sent for mass spectrometry analysis. Prepared BioID2 lysates were analyzed using TMT mass spectrometry. For each sample, four technical repeats were sent for analysis at the European Molecular Biology Laboratory (EMBL) Proteomics Core Facility. Samples were multiplexed in two batches, with two repeats of each experimental condition on each plate.

### Immunoprecipitation

Cells were seeded in 10-cm dishes at 1 × 10^6^ cells per dish for 2 days. Lysis buffer [1 M Tris (pH 7.5), 4 M NaCl, 10 mM Na_3_VO_4_, 1% NP-40 alternative, 0.1 M PMSF, 1x protease inhibitor, and 1 M DTT] was added directly to each plate, and cells were scraped before transfer to an Eppendorf tube on ice. After a 30-min incubation lysates were spun at 900g for 4 min at 4°C, and the supernatant was taken. Samples were precleared for 4 hours with the addition of 30 μl of PGS (protein G sepharose) on rotation at 4°C. One microgram of anti-SYCP1 antibody (Abcam, 15090) was added and incubated with rotation overnight at 4°C. PGS was added at 30 μl per tube for 1 hour at 4°C. Beads were pelleted by centrifugation, and the supernatant discarded. The beads were then washed with 1 ml of wash buffer A (PBS, 0.2% Triton X-100, and 350 mM NaCl) followed by centrifugation. The supernatant was removed and washed with 1 ml of wash buffer B (PBS and 0.2% Triton X-100) and then centrifuged again. The resultant supernatant had 40 μl of Laemmli with B-ME added and was boiled at 95°C for 10 min. The supernatant was run on an SDS–polyacrylamide gel electrophoresis gel and blotted using antibodies anti-NFX1 (Abcam, ab176733), anti-NFYA (Rockland, 100-401-100), anti-SP1 (Abcam, ab227383), anti-SP5 (Invitrogen, PA5-103505), anti-KLF3 (Abcam, ab154531), or anti-SP2 (Invitrogen, PA5-35984).

### COS-7 culture and transfection

COS-7 cells were maintained in DMEM supplemented with 10% (v/v) FCS and 100 μg/ml PenStrep (Invitrogen) at 37°C in 5% CO_2_. Transfections were performed using Lipofectamine 3000 reagent (Invitrogen) following the manufacturer’s instructions.

### Slide preparation and imaging

Cells expressing enhanced green fluorescent protein (EGFP)–SYCP1 were fixed 24 hours posttransfection in 100% methanol at −20°C for 20 min. After fixation, cells were washed three times for 5 min in PBST (1x PBS and 0.1% Triton-X100), stained with Hoechst (10 μg/ml) for 10 min, and washed once more in PBST. Coverslips were mounted on a slide with 10 μl of VECTASHIELD mounting media (Vector).

The slides were imaged on a Nikon TI2 inverted microscope (Nikon), equipped with a Photometrics Prime 95B camera and the Nikon Elements 5.1 software. The images were further processed in ImageJ.

### Cloning

The different SYCP1 constructs were cloned into the pEGFP-C3 vector (Clontech) following the in-house SLIC (Sequence and Ligation Independent Cloning) reaction protocol and transformed into chemically competent *Escherichia coli* (DH5α). Plasmid DNA was extracted from the bacterial cultures using the GeneJET Plasmid Miniprep Kit (Thermo Fisher Scientific) following the manufacturer’s instructions. All plasmids were sequenced before use.

### Chromatin immunoprecipitation and qPCR

ChIP-qPCR was performed to determine SP1, SP2, and SP5 binding at gene promoters in MCF7 WT and SYCP1 KO cell lines. Cells were seeded at 3.5 × 10^6^ in 2x 100-mm dish and cultured for 3 to 4 days. Cross-linking was performed with 1% formaldehyde for 7 min at room temperature, quenched with 0.125 M glycine, and washed with ice-cold PBS. Cells were scraped, pelleted, and either processed immediately or snap frozen at −80°C.

Chromatin was extracted through sequential lysis buffers and sonicated (30-s on, 20-s off, 10 cycles) to yield fragments of 400 to 600 bp, as confirmed on an agarose gel after RNase A and proteinase K treatment. For each ChIP, 80 μg of chromatin was incubated overnight at 4°C with 2 μg of antibody prebound to 40 μl of Dynabeads Protein G (Invitrogen, 10003D). Antibodies against SP1 (Abcam, ab227383), SP2 (Invitrogen, PA5-35984), and SP5 (Invitrogen, PA5-103505) were used, along with a nonimmunized, species matched IgG control. Beads were washed five times with ice-cold radioimmunoprecipitation assay (RIPA) buffer and once with tris-buffered saline (TBS). Chromatin was eluted and reverse cross-linked overnight at 65°C, followed by proteinase K treatment and purification using the GeneJet PCR purification kit (Thermo Fisher Scientific, K0701).

Candidate target genes were selected using the UCSC Genome Browser (*36*) for SP1, SP2, and SP5, based on regions with strong predicted promoter occupancy. These were then compared with genes identified by SYCP1 CUT&Tag to identify overlapping targets. Overlapping and unique targets for each TF were selected for qPCR analysis. Promoter regions 1000 bp upstream of the transcription start site were retrieved for each target gene. TF binding motifs were obtained from the JASPAR (*37*) database and mapped to promoter regions using FIMO (*38*). Primers were designed to amplify 100- to 200-bp regions encompassing the predicted binding motifs.

qPCR was performed using the PowerUp SYBR Green Master Mix (Applied Biosystems, A25742) on a standard real-time thermal cycler. Each ChIP condition was analyzed in three biologically independent replicates. Data were normalized to input DNA using the percentage input, and fold enrichment was calculated relative to IgG to correct for nonspecific binding. Statistical comparisons between WT and KO samples were made using the two-tailed unpaired Welches *t* test, with significance defined as *P* < 0.05.

### Statistics and reproducibility

All data were first tested for parametric distribution and analyzed with the GraphPad Prism software. Parametric data were analyzed using appropriate *t* tests or ANOVA with Bonferroni’s comparison test for multiple group comparisons. Nonparametric data were analyzed using the Mann-Whitney or Wilcoxon signed-rank test. By convention, ***P < 0.001, **P < 0.01, and *P < 0.05. The number of experiments is indicated for each figure where experiments with internal replicates are shown as ± SEM whereas experiments where this was not possible are shown as ± SD.

Survival analysis of patients with cancer from TCGA was performed on samples of primary tumors. The patients were ordered according to *SYCP1* expression, and the cutoff between the first and third quantiles was used to divide patients into *SYCP1* high- and low-expressing subgroups.

## Supplementary Materials

### This PDF file includes:

Figs. S1 to S11 Table S1 References

## References

[1] D. Hanahan, R. A. Weinberg, Hallmarks of cancer: The next generation. Cell 144, 646–674 (2011). 21376230 10.1016/j.cell.2011.02.013

[2] J. Fraune, S. Schramm, M. Alsheimer, R. Benavente, S. Schramm, M. Alsheimer, R. Benavente, The mammalian synaptonemal complex: Protein components, assembly and role in meiotic recombination. Exp. Cell Res. 318, 1340–1346 (2012). 22394509 10.1016/j.yexcr.2012.02.018

[3] A. Geisinger, R. Benavente, Mutations in genes coding for synaptonemal complex proteins and their impact on human fertility. Cytogenet. Genome Res. 150, 77–85 (2017). 10.1159/000453344 27997882

[4] I. F. Sou, G. Hamer, W.-W. Tee, G. Vader, U. L. McClurg, Cancer and meiotic gene expression: Two sides of the same coin? Curr. Top. Dev. Biol. 151, 43–68 (2023). 36681477 10.1016/bs.ctdb.2022.06.002

[5] N. Hosoya, K. Miyagawa, Synaptonemal complex proteins modulate the level of genome integrity in cancers. Cancer Sci. 112, 989–996 (2021). 33382503 10.1111/cas.14791 PMC7935773

[6] N. Hosoya, M. Okajima, A. Kinomura, Y. Fujii, T. Hiyama, J. Sun, S. Tashiro, K. Miyagawa, Synaptonemal complex protein SYCP3 impairs mitotic recombination by interfering with BRCA2. EMBO Rep. 13, 44–51 (2011). 22116401 10.1038/embor.2011.221 PMC3246250

[7] N. Hosoya, M. Ono, K. Miyagawa, Somatic role of SYCE2: An insulator that dissociates HP1α from H3K9me3 and potentiates DNA repair. Life Sci. Alliance 1, e201800021 (2018). 30456351 10.26508/lsa.201800021 PMC6238414

[8] S. Sandhu, I. F. Sou, J. E. Hunter, L. Salmon, C. L. Wilson, N. D. Perkins, N. Hunter, O. R. Davies, U. L. McClurg, Centrosome dysfunction associated with somatic expression of the synaptonemal complex protein TEX12. Commun. Biol. 4, 1371 (2021). 34880391 10.1038/s42003-021-02887-4 PMC8654964

[9] Y. Wang, B. Gao, L. Zhang, X. Wang, X. Zhu, H. Yang, F. Zhang, X. Zhu, B. Zhou, S. Yao, A. Nagayama, S. Lee, J. Ouyang, S. B. Koh, E. L. Eisenhauer, D. Zarrella, K. Lu, B. R. Rueda, L. Zou, X. A. Su, O. Yeku, L. W. Ellisen, X. S. Wang, L. Lan, Meiotic protein SYCP2 confers resistance to DNA-damaging agents through R-loop-mediated DNA repair. Nat. Commun. 15, 1568 (2024). 38383600 10.1038/s41467-024-45693-2 PMC10881575

[10] R. L. Meuwissen, H. H. Offenberg, A. J. Dietrich, A. Riesewijk, M. van Iersel, C. Heyting, A coiled-coil related protein specific for synapsed regions of meiotic prophase chromosomes. EMBO J. 11, 5091–5100 (1992). 1464329 10.1002/j.1460-2075.1992.tb05616.x PMC556987

[11] K. K. Billmyre, E. A. Kesler, D. Tsuchiya, T. J. Corbin, K. Weaver, A. Moran, Z. Yu, L. Adams, K. Delventhal, M. Durnin, O. R. Davies, R. S. Hawley, SYCP1 head-to-head assembly is required for chromosome synapsis in mouse meiosis. Sci. Adv. 9, eadi1562 (2023). 37862414 10.1126/sciadv.adi1562 PMC10588951

[12] M. Kalejs, A. Ivanov, G. Plakhins, M. S. Cragg, D. Emzinsh, T. M. Illidge, J. Erenpreisa, Upregulation of meiosis-specific genes in lymphoma cell lines following genotoxic insult and induction of mitotic catastrophe. BMC Cancer 6, 6 (2006). 16401344 10.1186/1471-2407-6-6 PMC1351196

[13] S. H. Lim, S. Austin, E. Owen-Jones, L. Robinson, Expression of testicular genes in haematological malignancies. Br. J. Cancer 81, 1162–1164 (1999). 10584877 10.1038/sj.bjc.6690824 PMC2374325

[14] I. V. Litvinov, E. Netchiporouk, B. Cordeiro, H. Zargham, K. Pehr, M. Gilbert, Y. Zhou, L. Moreau, A. Woetmann, N. Ødum, T. S. Kupper, D. Sasseville, Ectopic expression of embryonic stem cell and other developmental genes in cutaneous T-cell lymphoma. Oncoimmunology 3, e970025 (2014). 25941598 10.4161/21624011.2014.970025 PMC4368148

[15] I. V. Litvinov, E. Netchiporouk, B. Cordeiro, M. A. Doré, L. Moreau, K. Pehr, M. Gilbert, Y. Zhou, D. Sasseville, T. S. Kupper, The use of transcriptional profiling to improve personalized diagnosis and management of cutaneous T-cell lymphoma (CTCL). Clin. Cancer Res. 21, 2820–2829 (2015). 25779945 10.1158/1078-0432.CCR-14-3322 PMC4470792

[16] P. Niemeyer, O. Türeci, T. Eberle, N. Graf, M. Pfreundschuh, U. Sahin, Expression of serologically identified tumor antigens in acute leukemias. Leuk. Res. 27, 655–660 (2003). 12681366 10.1016/s0145-2126(02)00230-8

[17] S. M. Oba-Shinjo, O. L. Caballero, A. A. Jungbluth, S. Rosemberg, L. J. Old, A. J. Simpson, S. K. Marie, Cancer-testis (CT) antigen expression in medulloblastoma. Cancer Immun. 8, 7 (2008). 18426187 PMC2935780

[18] D. I. Vodolazhsky, D. S. Kutilin, K. A. Mogushkova, O. I. Kit, Specific features of transcription activity of cancer-testis antigens in patients with metastatic and non-metastatic breast cancer. Bull. Exp. Biol. Med. 165, 382–385 (2018). 30006881 10.1007/s10517-018-4175-x

[19] C. Zhang, T. Kawakami, Y. Okada, K. Okamoto, Distinctive epigenetic phenotype of cancer testis antigen genes among seminomatous and nonseminomatous testicular germ-cell tumors. Genes Chromosomes Cancer 43, 104–112 (2005). 15672408 10.1002/gcc.20160

[20] S. H. Payne, The utility of protein and mRNA correlation. Trends Biochem. Sci. 40, 1–3 (2015). 25467744 10.1016/j.tibs.2014.10.010 PMC4776753

[21] J. Li, Y. Zhang, C. Yang, R. Rong, Discrepant mRNA and protein expression in immune cells. Curr. Genomics 21, 560–563 (2020). 33414677 10.2174/1389202921999200716103758 PMC7770634

[22] H. S. Kaya-Okur, S. J. Wu, C. A. Codomo, E. S. Pledger, T. D. Bryson, J. G. Henikoff, K. Ahmad, S. Henikoff, CUT&Tag for efficient epigenomic profiling of small samples and single cells. Nat. Commun. 10, 1930 (2019). 31036827 10.1038/s41467-019-09982-5 PMC6488672

[23] R. W. Robey, C. M. Fitzsimmons, W. M. Guiblet, W. J. E. Frye, J. M. González Dalmasy, L. Wang, D. A. Russell, L. M. Huff, A. J. Perciaccante, F. Ali-Rahmani, C. C. Lipsey, H. M. Wade, A. V. Mitchell, S. S. Maligireddy, D. Terrero, D. Butcher, E. F. Edmondson, L. M. Jenkins, T. Nikitina, V. B. Zhurkin, A. K. Tiwari, A. D. Piscopio, R. A. Totah, S. E. Bates, H. E. Arda, M. M. Gottesman, P. J. Batista, The methyltransferases METTL7A and METTL7B confer resistance to thiol-based histone deacetylase inhibitors. Mol. Cancer Ther. 23, 464–477 (2024). 38151817 10.1158/1535-7163.MCT-23-0144 PMC11223745

[24] J. M. Dunce, O. M. Dunne, M. Ratcliff, C. Millán, S. Madgwick, I. Usón, O. R. Davies, Structural basis of meiotic chromosome synapsis through SYCP1 self-assembly. Nat. Struct. Mol. Biol. 25, 557–569 (2018). 29915389 10.1038/s41594-018-0078-9 PMC6606445

[25] U. L. McClurg, A. Nabbi, C. Ricordel, S. Korolchuk, S. McCracken, R. Heer, L. Wilson, L. M. Butler, B. K. Irving-Hooper, R. Pedeux, C. N. Robson, K. T. Riabowol, O. Binda, Human ex vivo prostate tissue model system identifies ING3 as an oncoprotein. Br. J. Cancer 118, 713–726 (2018). 29381681 10.1038/bjc.2017.447 PMC5846061

[26] A. E. P. Loftus, M. S. Romano, A. N. Phuong, B. J. McKinnel, M. T. Muir, M. Furqan, J. C. Dawson, L. Avalle, A. T. Douglas, R. L. Mort, A. Byron, N. O. Carragher, S. M. Pollard, V. G. Brunton, M. C. Frame, An ILK/STAT3 pathway controls glioblastoma stem cell plasticity. Dev. Cell 59, 3197–3212.e7 (2024). 39326421 10.1016/j.devcel.2024.09.003

[27] B. Li, C. N. Dewey, RSEM: Accurate transcript quantification from RNA-Seq data with or without a reference genome. BMC Bioinformatics 12, 323 (2011). 21816040 10.1186/1471-2105-12-323 PMC3163565

[28] M. I. Love, W. Huber, S. Anders, Moderated estimation of fold change and dispersion for RNA-seq data with DESeq2. Genome Biol. 15, 550 (2014). 25516281 10.1186/s13059-014-0550-8 PMC4302049

[29] F. Ramírez, F. Dündar, S. Diehl, B. A. Grüning, T. Manke, deepTools: A flexible platform for exploring deep-sequencing data. Nucleic Acids Res. 42, W187–W191 (2014). 24799436 10.1093/nar/gku365 PMC4086134

[30] A. R. Quinlan, BEDTools: The Swiss-Army tool for genome feature analysis. Curr. Protoc. Bioinformatics 47, 11.12.1–11.12.34 (2014). 10.1002/0471250953.bi1112s47 PMC4213956 25199790

[31] Y. Zhang, T. Liu, C. A. Meyer, J. Eeckhoute, D. S. Johnson, B. E. Bernstein, C. Nusbaum, R. M. Myers, M. Brown, W. Li, X. S. Liu, Model-based analysis of ChIP-Seq (MACS). Genome Biol. 9, R137 (2008). 18798982 10.1186/gb-2008-9-9-r137 PMC2592715

[32] Q. Wang, M. Li, T. Wu, L. Zhan, L. Li, M. Chen, W. Xie, Z. Xie, E. Hu, S. Xu, G. Yu, Exploring epigenomic datasets by ChIPseeker. Curr. Protoc. 2, e585 (2022). 36286622 10.1002/cpz1.585

[33] S. Heinz, C. Benner, N. Spann, E. Bertolino, Y. C. Lin, P. Laslo, J. X. Cheng, C. Murre, H. Singh, C. K. Glass, Simple combinations of lineage-determining transcription factors prime cis-regulatory elements required for macrophage and B cell identities. Mol. Cell 38, 576–589 (2010). 20513432 10.1016/j.molcel.2010.05.004 PMC2898526

[34] A. Kiesel, C. Roth, W. Ge, M. Wess, M. Meier, J. Söding, The BaMM web server for de-novo motif discovery and regulatory sequence analysis. Nucleic Acids Res. 46, W215–W220 (2018). 29846656 10.1093/nar/gky431 PMC6030882

[35] A. Dobin, C. A. Davis, F. Schlesinger, J. Drenkow, C. Zaleski, S. Jha, P. Batut, M. Chaisson, T. R. Gingeras, STAR: Ultrafast universal RNA-seq aligner. Bioinformatics 29, 15–21 (2013). 23104886 10.1093/bioinformatics/bts635 PMC3530905

[36] G. Perez, G. P. Barber, A. Benet-Pages, J. Casper, H. Clawson, M. Diekhans, C. Fischer, J. N. Gonzalez, A. S. Hinrichs, C. M. Lee, L. R. Nassar, B. J. Raney, M. L. Speir, M. J. van Baren, C. J. Vaske, D. Haussler, W. J. Kent, M. Haeussler, The UCSC Genome Browser database: 2025 Update. Nucleic Acids Res. 53, D1243–D1249 (2025). 39460617 10.1093/nar/gkae974 PMC11701590

[37] I. Rauluseviciute, R. Riudavets-Puig, R. Blanc-Mathieu, J. A. Castro-Mondragon, K. Ferenc, V. Kumar, R. B. Lemma, J. Lucas, J. Chèneby, D. Baranasic, A. Khan, O. Fornes, S. Gundersen, M. Johansen, E. Hovig, B. Lenhard, A. Sandelin, W. W. Wasserman, F. Parcy, A. Mathelier, JASPAR 2024: 20th Anniversary of the open-access database of transcription factor binding profiles. Nucleic Acids Res. 52, D174–D182 (2024). 37962376 10.1093/nar/gkad1059 PMC10767809

[38] C. E. Grant, T. L. Bailey, W. S. Noble, FIMO: Scanning for occurrences of a given motif. Bioinformatics 27, 1017–1018 (2011). 21330290 10.1093/bioinformatics/btr064 PMC3065696

[39] A. Liberzon, C. Birger, H. Thorvaldsdóttir, M. Ghandi, J. P. Mesirov, P. Tamayo, The Molecular Signatures Database hallmark gene set collection. Cell Syst. 1, 417–425 (2015). 26771021 10.1016/j.cels.2015.12.004 PMC4707969

[40] A. Subramanian, P. Tamayo, V. K. Mootha, S. Mukherjee, B. L. Ebert, M. A. Gillette, A. Paulovich, S. L. Pomeroy, T. R. Golub, E. S. Lander, J. P. Mesirov, Gene set enrichment analysis: A knowledge-based approach for interpreting genome-wide expression profiles. Proc. Natl. Acad. Sci. U.S.A. 102, 15545–15550 (2005). 16199517 10.1073/pnas.0506580102 PMC1239896

[41] L. Berglund, E. Björling, P. Oksvold, L. Fagerberg, A. Asplund, C. A. Szigyarto, A. Persson, J. Ottosson, H. Wernérus, P. Nilsson, E. Lundberg, A. Sivertsson, S. Navani, K. Wester, C. Kampf, S. Hober, F. Pontén, M. Uhlén, A genecentric human protein atlas for expression profiles based on antibodies. Mol. Cell. Proteomics 7, 2019–2027 (2008). 18669619 10.1074/mcp.R800013-MCP200

