# Supplementary material for: Moonlighting role of meiotic SYCP1 in breast cancer: A chromatin-bound regulator of DNA repair, transcription, and drug resistance
Source: Sci Adv. 2026 Jul 8;12(28):eaea2067. doi: 10.1126/sciadv.aea2067 (PMC13344282; doi:10.1126/sciadv.aea2067)
Supplement: Supplementary file 1 — Figs. S1 to S11 Table S1 References [file sciadv.aea2067_sm.pdf]

Science Advances
AAAS

Supplementary Materials for

Moonlighting role of meiotic SYCP1 in breast cancer: A chromatin-bound regulator of DNA repair, transcription, and drug resistance

Louise C. Brennan *et al.*

Corresponding author: Wee-Wei Tee, Tee_Wee_Wei@a-star.edu.sg;
Urszula L. McClurg, urszula.mcclurg@liverpool.ac.uk

Sci. Adv. **12**, eaea2067 (2026)
DOI: 10.1126/sciadv.aea2067

This PDF file includes:

Figs. S1 to S11
Table S1
References

Supplementary figures

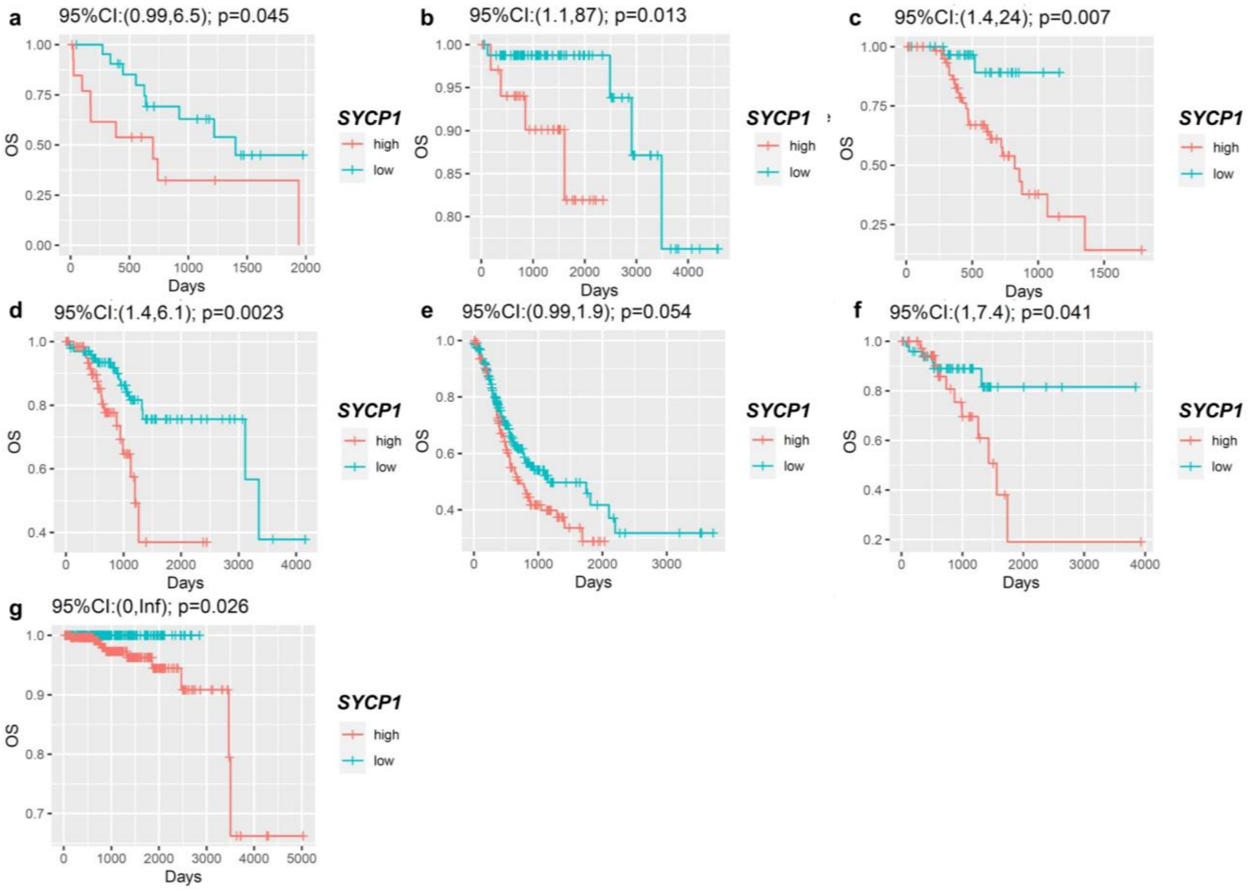

Supplementary Figure 1 TCGA patients with high SYCP1 expression have shorter overall survival (**a**) cholangiocarcinoma HR 2.5, (**b**) thymoma HR 9.7, (**c**) skin cutaneous melanoma HR 5.8, (**d**) uterine corpus endometrial carcinoma HR 3, (**e**) stomach adenocarcinoma HR 1.4, (**f**) rectal adenocarcinoma HR2.7. p-value was calculated by log-rank test. HR: hazard ratio, (**g**) prostate adenocarcinoma HR 4.2^{e+08}

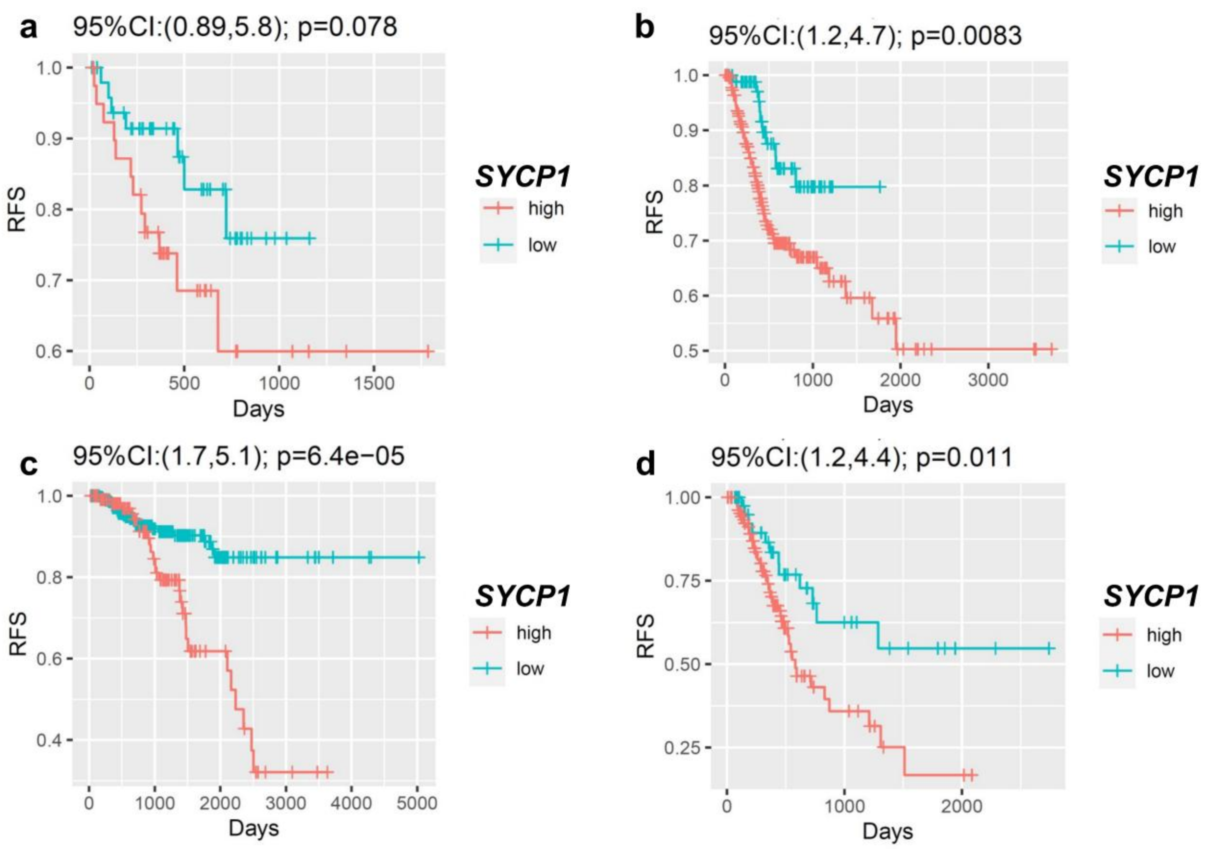

Supplementary Figure 2 TCGA patients with high SYCP1 expression have shorter relapse free survival (**a**) skin cutaneous melanoma HR 2.3, (**b**) stomach adenocarcinoma HR 2.4, (**c**) prostate adenocarcinoma HR 2.9, (**d**) pancreatic adenocarcinoma HR 2.3. p-value was calculated by log-rank test. HR: hazard ratio.

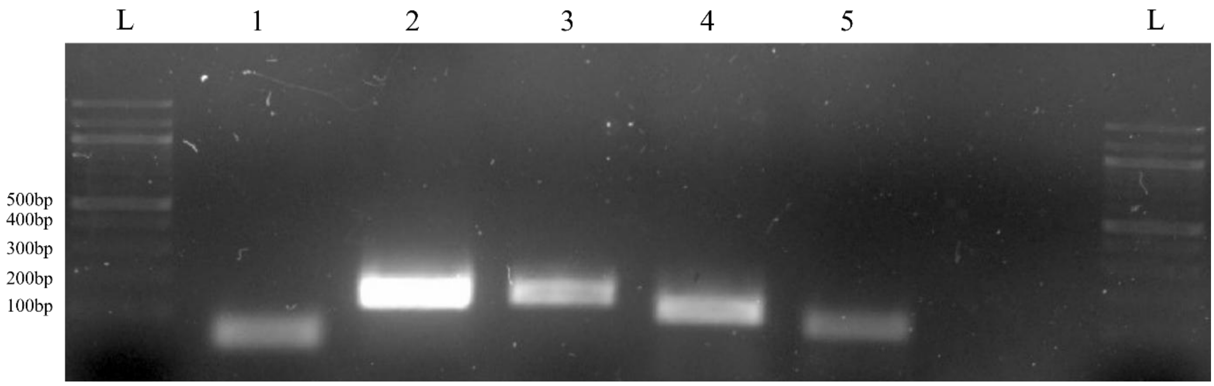

Supplementary Figure 3 *Agarose gel electrophoresis of qRT-PCR products from MCF7 cDNA, for determining specificity of primers L: DNA ladder; 1: HRPT1; 2: SYCP1; 3: POLD1; 4: MCM4; 5: PLK.*

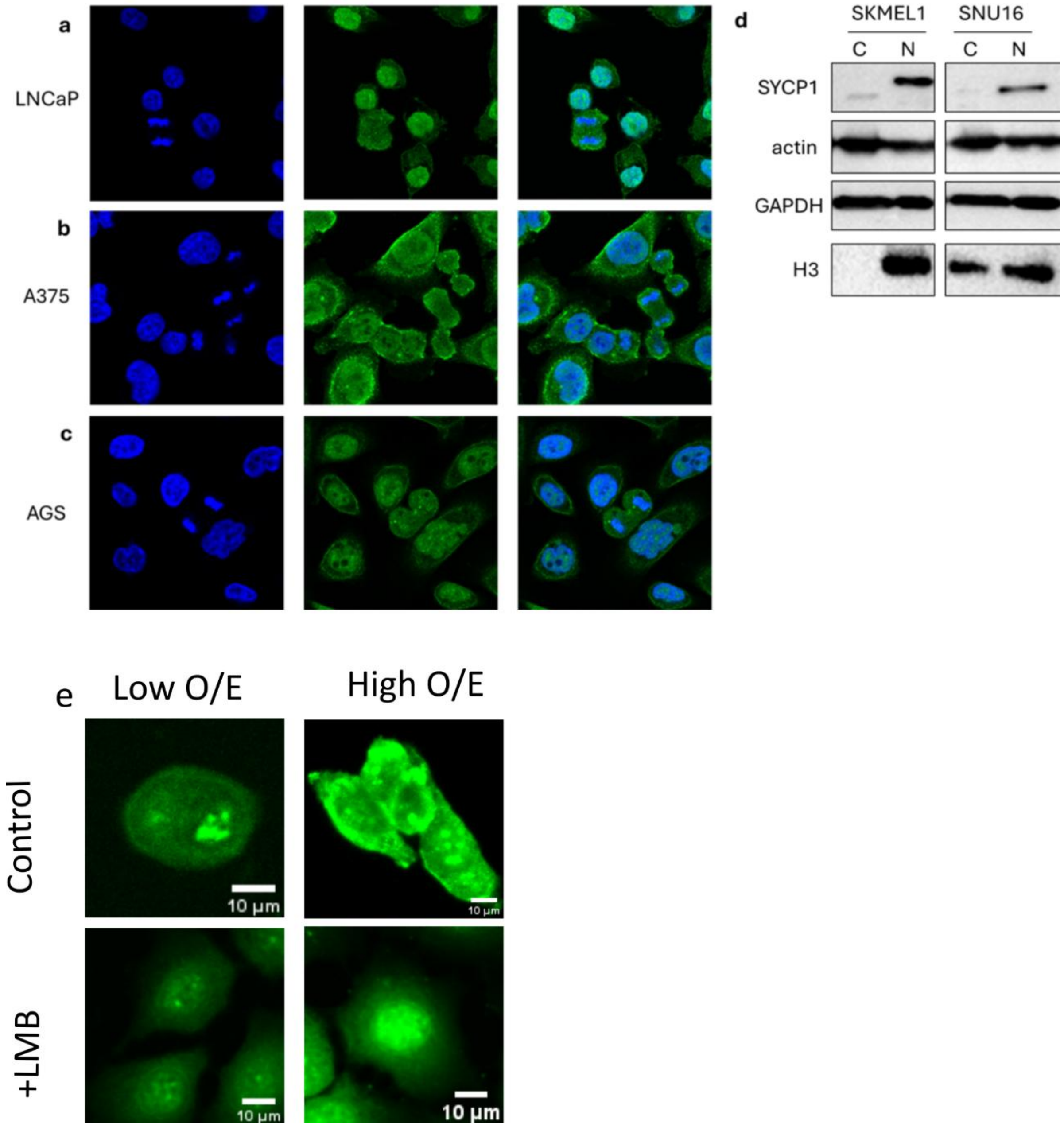

Supplementary Figure 4 SYCP1 is a nuclear protein in cancer (**a**) prostate cancer LNCaP cells, (**b**) stomach cancer A375 cells, (**c**) stomach cancer AGS cells. SYCP1 immunolabelled in green with DNA DAPI stained in blue, (**d**) cell fractionation of SKMEL1 melanoma cells and SNU16 stomach cancer cells. (**e**) SYCP1-eGFP overexpression in MCF7 cells SYCP1 is consistently retained in the nucleus upon leptomycin B treatment.

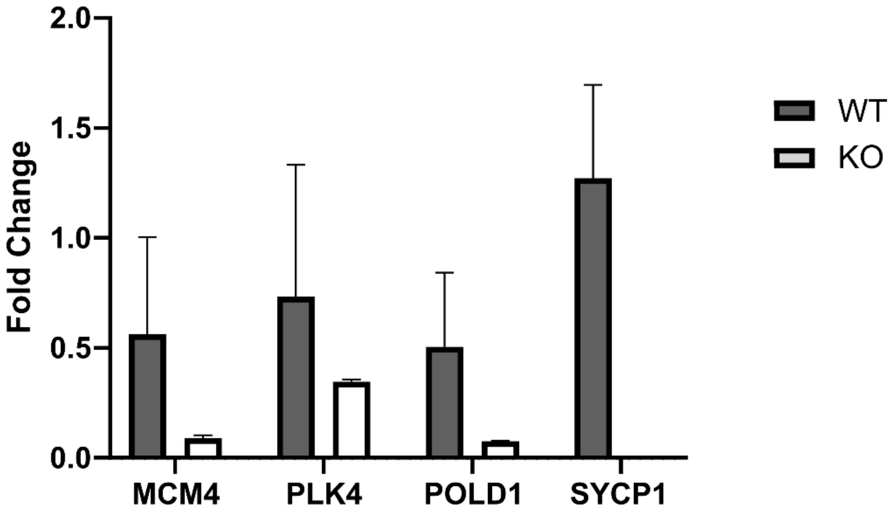

Supplementary Figure 5 validation of hits from RNAseq. *RT-qPCR was performed on cDNA extracted from MCF7 and MCF7 SYCP1 KO cells, values were normalised to HPRT1 housekeeping and fold change was calculated compared to a repeat of MCF7 WT. Error bars represent SEM, n=2.*

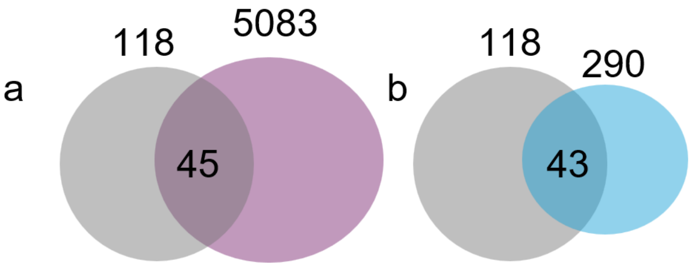

c

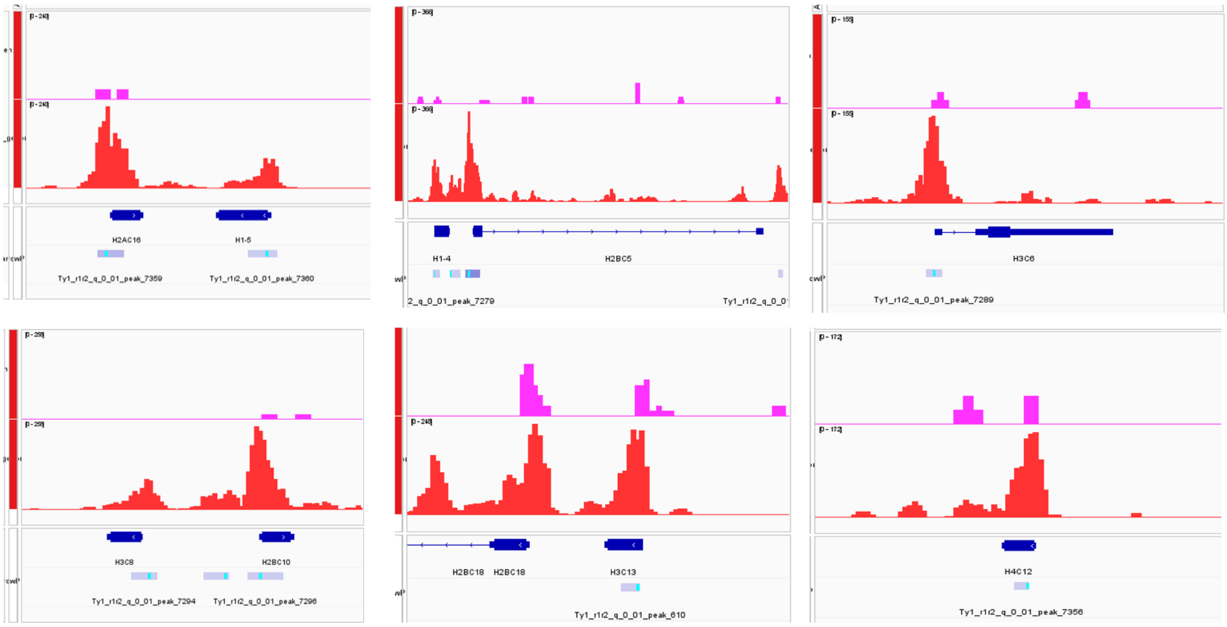

Supplementary figure 6 SYCP1 preferentially binds to promoters and regulates expression of histone genes. *(**a**) comparative analysis of 5083 genes with SYCP1 promoter binding vs histone genes using all histone genes from HUGO, Parameters: 45, 5083, 118, 20000; expected number of successes = 29.9; results are over enriched 1.5 fold compared to expectations; hypergeometric p-value = 0.0015. (**b**) comparative analysis of 290 genes with SYCP1 promoter binding that were also significantly downregulated upon SYCP1 knock-out vs histone genes using all histone genes from HUGO* (https://www.genenames.org/download/custom/)*,; results are over enriched 26.3 fold compared to expectations; hypergeometric p-value = 1.8e-52. (**c**) SYCP1 histone peaks.*

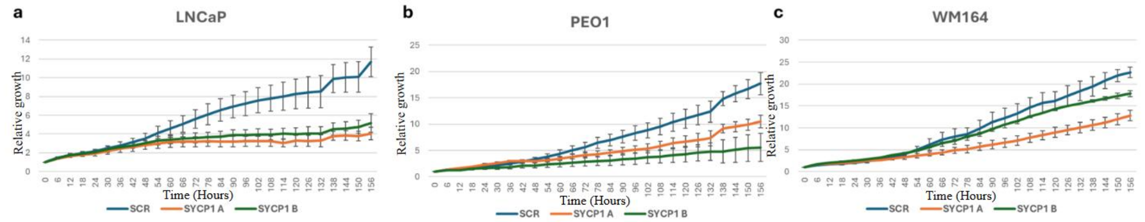

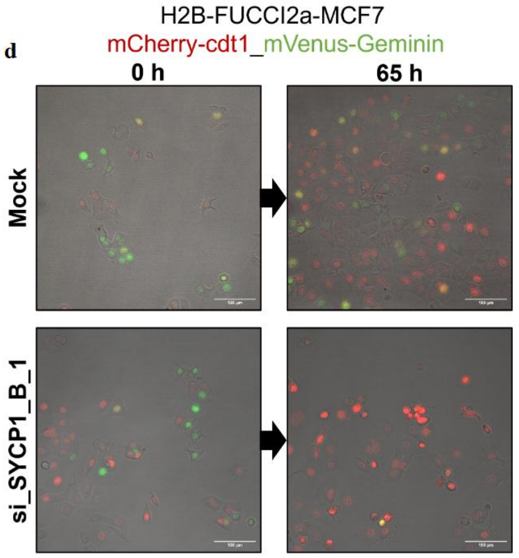

Supplementary figure 7 SYCP1 silencing abrogates cancer cell growth. (**a-c**) Cells were seeded with non-targeting siRNA (SCR) and two SYCP1 targeting sequences (SYCP1 A and B). Cell growth was monitored using incucyte for 156hrs and is presented as fold change over control SCR treated cells. (**a**) LNCaP prostate cancer cells, (**b**) PEO1 ovarian cancer cells, (**c**) WM164 melanoma cells. Error bars represent SD. (**d**) MCF7 cells stably transfected with H2B-Fucci2a were transfected with control (SCR), SYCP1 targeting siRNAs or a mock transfection. 24 hours post transfection, live cell imaging was initiated, with images acquired every 15 minutes for 65 hours. Images show fields of view at 0 and 65 hours, for Mock and siSYCP1_B transfections.

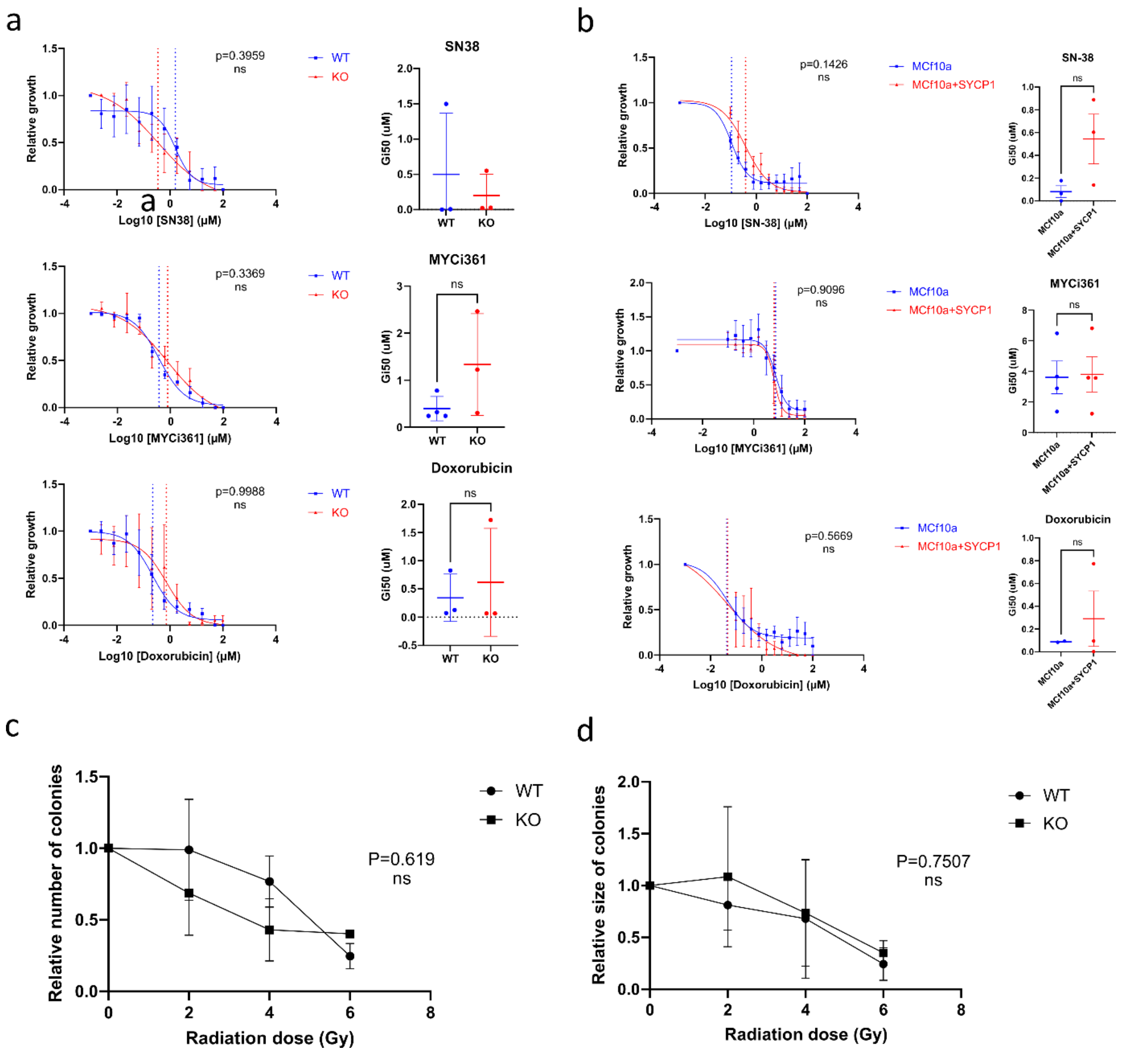

Supplementary Figure 8. SYCP1 effect on response of some cancer therapeutics. *(**a**) Gi50s of WT and SYCP1 KO MCF7 cells in response to SN-38, MYCi361 and Doxorubicin. (**b**) Gi50s of MCF10a cells and MCF10a transfected with SYCP1 in response to SN-38, MYCi361 and Doxorubicin. (**c**) Relative number of colonies in a colony formation assay after varying doses of radiation, in WT and SYCP1 KO MCF7 cells. (**e**) Relative size of colonies after varying doses of radiation, in WT and SYCP1 KO MCF7 cells. In all subfigures error bars represent SEM.*

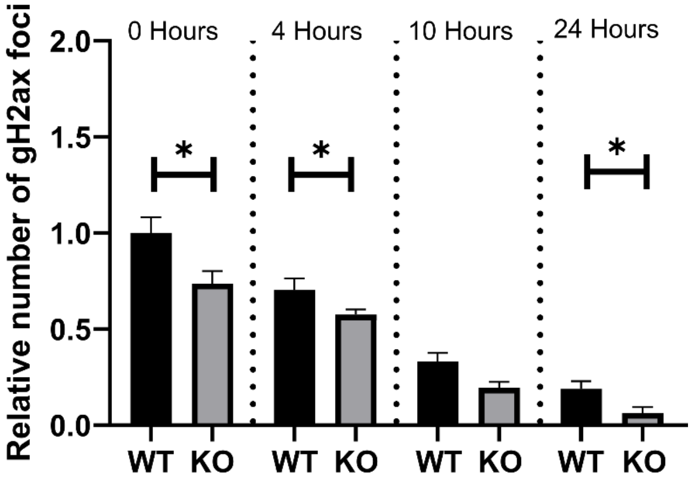

Supplementary Figure 9 Relative number of gH2ax foci after DNA damage recovery*. gH2ax foci were quantified in WT and SYCP1 KO MCF7 cells after Cisplatin treatment, over 24 hours. 15 cells were quantified for each condition and timepoint. Error bars represent SEM.*

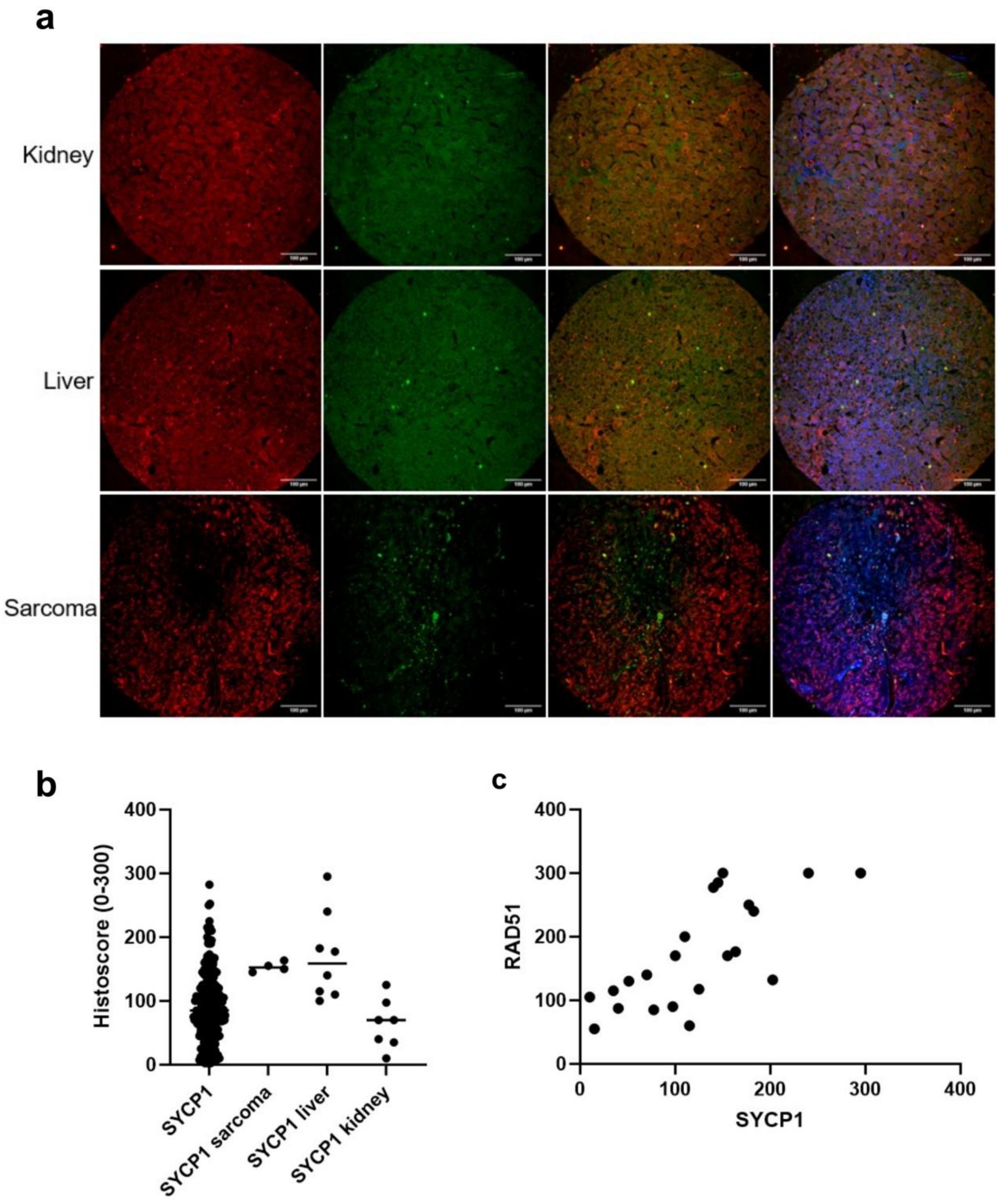

Supplementary Figure 10 SYCP1 colocalises with RAD51 in kidney, liver and sarcoma patients. *(**a**) TMAs from kidney, liver and sarcoma cancer patients were stained for both RAD51(green) and SYCP1 (red) (**b**) SYCP1 histoscore distribution by cancer type.(**c**) RAD51 and SYCP1 have a positive correlation in the cohort (p<0.0001).*

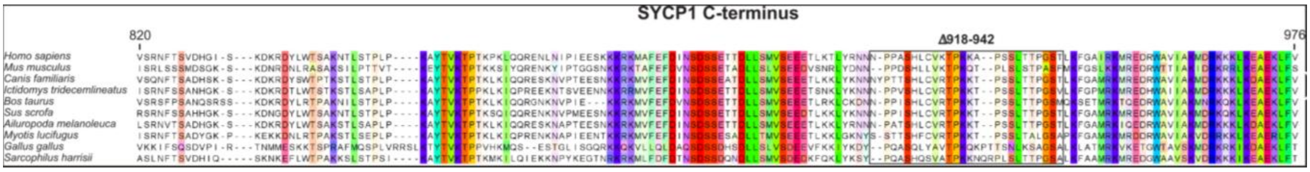

Supplementary Figure 11 SYCP1 C-terminus conservation alignment.

	**Transcript**	**Protein**			
**Cell type**	**nPCM**	**Female 1**	**Female 2**	**Female 3**	**Female 4**
Breast myoepithelial cells	0	0	0	0	0
Breast secretory cells	0.3	0	0	0	0
Breast lactating cells	6	0	0	0	0
Breast hormone-responsive cells	0.1	0	0	0	0
Vascular endothelial cells	0.2	0	0	0	0
Lymphatic endothelial cells	0	0	0	0	0
Pericytes	0	0	0	0	0
Vascular smooth muscle cells	0.7	0	0	0	0
Fibroblasts	0	0	0	0	0
Monocytes	0	0	0	0	0
Macrophages	0	0	0	0	0
T-cells	0.1	0	0	0	0
Innate lymphoid cells	0	0	0	0	0
B-cells	0	0	0	0	0
Plasma cells	0	0	0	0	0
CDC	0	0	0	0	0
PDCs	0	0	0	0	0

Supplementary Table 1 SYCP1protein is absent in normal breast tissue. *Breast tissue from 4 patients with no breast cancer age 38, 47, 52 and 80 representing a spread of reproductive age and menopause status was analysed by immunohistochemistry and quantified. Similarly single cell RNAseq of normal breast tissue was performed and analysed. SYCP1 transcript was detected at low levels in distinct cell types however, no SYCP1 protein was detected in any of the study subjects. Samples from (41).*

REFERENCES

1. D. Hanahan, R. A. Weinberg, Hallmarks of cancer: The next generation. *Cell* **144**, 646–674 (2011).

2. J. Fraune, S. Schramm, M. Alsheimer, R. Benavente, S. Schramm, M. Alsheimer, R. Benavente, The mammalian synaptonemal complex: Protein components, assembly and role in meiotic recombination. *Exp. Cell Res.* **318**, 1340–1346 (2012).

3. A. Geisinger, R. Benavente, Mutations in genes coding for synaptonemal complex proteins and their impact on human fertility. *Cytogenet. Genome Res.* **150**, 77–85 (2017).

4. I. F. Sou, G. Hamer, W.-W. Tee, G. Vader, U. L. McClurg, Cancer and meiotic gene expression: Two sides of the same coin? *Curr. Top. Dev. Biol.* **151**, 43–68 (2023).

5. N. Hosoya, K. Miyagawa, Synaptonemal complex proteins modulate the level of genome integrity in cancers. *Cancer Sci.* **112**, 989–996 (2021).

6. N. Hosoya, M. Okajima, A. Kinomura, Y. Fujii, T. Hiyama, J. Sun, S. Tashiro, K. Miyagawa, Synaptonemal complex protein SYCP3 impairs mitotic recombination by interfering with BRCA2. *EMBO Rep.* **13**, 44–51 (2011).

7. N. Hosoya, M. Ono, K. Miyagawa, Somatic role of SYCE2: An insulator that dissociates HP1α from H3K9me3 and potentiates DNA repair. *Life Sci. Alliance* **1**, e201800021 (2018).

8. S. Sandhu, I. F. Sou, J. E. Hunter, L. Salmon, C. L. Wilson, N. D. Perkins, N. Hunter, O. R. Davies, U. L. McClurg, Centrosome dysfunction associated with somatic expression of the synaptonemal complex protein TEX12. *Commun. Biol.* **4**, 1371 (2021).

9. Y. Wang, B. Gao, L. Zhang, X. Wang, X. Zhu, H. Yang, F. Zhang, X. Zhu, B. Zhou, S. Yao, A. Nagayama, S. Lee, J. Ouyang, S. B. Koh, E. L. Eisenhauer, D. Zarrella, K. Lu, B. R. Rueda, L. Zou, X. A. Su, O. Yeku, L. W. Ellisen, X. S. Wang, L. Lan, Meiotic protein SYCP2 confers resistance to DNA-damaging agents through R-loop-mediated DNA repair. *Nat. Commun.* **15**, 1568 (2024).

10. R. L. Meuwissen, H. H. Offenberg, A. J. Dietrich, A. Riesewijk, M. van Iersel, C. Heyting, A coiled-coil related protein specific for synapsed regions of meiotic prophase chromosomes. *EMBO J.* **11**, 5091–5100 (1992).

11. K. K. Billmyre, E. A. Kesler, D. Tsuchiya, T. J. Corbin, K. Weaver, A. Moran, Z. Yu, L. Adams, K. Delventhal, M. Durnin, O. R. Davies, R. S. Hawley, SYCP1 head-to-head assembly is required for chromosome synapsis in mouse meiosis. *Sci. Adv.* **9**, eadi1562 (2023).

12. M. Kalejs, A. Ivanov, G. Plakhins, M. S. Cragg, D. Emzinsh, T. M. Illidge, J. Erenpreisa, Upregulation of meiosis-specific genes in lymphoma cell lines following genotoxic insult and induction of mitotic catastrophe. *BMC Cancer* **6**, 6 (2006).

13. S. H. Lim, S. Austin, E. Owen-Jones, L. Robinson, Expression of testicular genes in haematological malignancies. *Br. J. Cancer* **81**, 1162–1164 (1999).

14. I. V. Litvinov, E. Netchiporouk, B. Cordeiro, H. Zargham, K. Pehr, M. Gilbert, Y. Zhou, L. Moreau, A. Woetmann, N. Ødum, T. S. Kupper, D. Sasseville, Ectopic expression of embryonic stem cell and other developmental genes in cutaneous T-cell lymphoma. *Oncoimmunology* **3**, e970025 (2014).

15. I. V. Litvinov, E. Netchiporouk, B. Cordeiro, M. A. Doré, L. Moreau, K. Pehr, M. Gilbert, Y. Zhou, D. Sasseville, T. S. Kupper, The use of transcriptional profiling to improve personalized diagnosis and management of cutaneous T-cell lymphoma (CTCL). *Clin. Cancer Res.* **21**, 2820–2829 (2015).

16. P. Niemeyer, O. Türeci, T. Eberle, N. Graf, M. Pfreundschuh, U. Sahin, Expression of serologically identified tumor antigens in acute leukemias. *Leuk. Res.* **27**, 655–660 (2003).

17. S. M. Oba-Shinjo, O. L. Caballero, A. A. Jungbluth, S. Rosemberg, L. J. Old, A. J. Simpson, S. K. Marie, Cancer-testis (CT) antigen expression in medulloblastoma. *Cancer Immun.* **8**, 7 (2008).

18. D. I. Vodolazhsky, D. S. Kutilin, K. A. Mogushkova, O. I. Kit, Specific features of transcription activity of cancer-testis antigens in patients with metastatic and non-metastatic breast cancer. *Bull. Exp. Biol. Med.* **165**, 382–385 (2018).

19. C. Zhang, T. Kawakami, Y. Okada, K. Okamoto, Distinctive epigenetic phenotype of cancer testis antigen genes among seminomatous and nonseminomatous testicular germ-cell tumors. *Genes Chromosomes Cancer* **43**, 104–112 (2005).

20. S. H. Payne, The utility of protein and mRNA correlation. *Trends Biochem. Sci.* **40**, 1–3 (2015).

21. J. Li, Y. Zhang, C. Yang, R. Rong, Discrepant mRNA and protein expression in immune cells. *Curr. Genomics* **21**, 560–563 (2020).

22. H. S. Kaya-Okur, S. J. Wu, C. A. Codomo, E. S. Pledger, T. D. Bryson, J. G. Henikoff, K. Ahmad, S. Henikoff, CUT&Tag for efficient epigenomic profiling of small samples and single cells. *Nat. Commun.* **10**, 1930 (2019).

23. R. W. Robey, C. M. Fitzsimmons, W. M. Guiblet, W. J. E. Frye, J. M. González Dalmasy, L. Wang, D. A. Russell, L. M. Huff, A. J. Perciaccante, F. Ali-Rahmani, C. C. Lipsey, H. M. Wade, A. V. Mitchell, S. S. Maligireddy, D. Terrero, D. Butcher, E. F. Edmondson, L. M. Jenkins, T. Nikitina, V. B. Zhurkin, A. K. Tiwari, A. D. Piscopio, R. A. Totah, S. E. Bates, H. E. Arda, M. M. Gottesman, P. J. Batista, The methyltransferases METTL7A and METTL7B confer resistance to thiol-based histone deacetylase inhibitors. *Mol. Cancer Ther.* **23**, 464–477 (2024).

24. J. M. Dunce, O. M. Dunne, M. Ratcliff, C. Millán, S. Madgwick, I. Usón, O. R. Davies, Structural basis of meiotic chromosome synapsis through SYCP1 self-assembly. *Nat. Struct. Mol. Biol.* **25**, 557–569 (2018).

25. U. L. McClurg, A. Nabbi, C. Ricordel, S. Korolchuk, S. McCracken, R. Heer, L. Wilson, L. M. Butler, B. K. Irving-Hooper, R. Pedeux, C. N. Robson, K. T. Riabowol, O. Binda, Human ex vivo prostate tissue model system identifies ING3 as an oncoprotein. *Br. J. Cancer* **118**, 713–726 (2018).

26. A. E. P. Loftus, M. S. Romano, A. N. Phuong, B. J. McKinnel, M. T. Muir, M. Furqan, J. C. Dawson, L. Avalle, A. T. Douglas, R. L. Mort, A. Byron, N. O. Carragher, S. M. Pollard, V. G. Brunton, M. C. Frame, An ILK/STAT3 pathway controls glioblastoma stem cell plasticity. *Dev. Cell* **59**, 3197–3212.e7 (2024).

27. B. Li, C. N. Dewey, RSEM: Accurate transcript quantification from RNA-Seq data with or without a reference genome. *BMC Bioinformatics* **12**, 323 (2011).

28. M. I. Love, W. Huber, S. Anders, Moderated estimation of fold change and dispersion for RNA-seq data with DESeq2. *Genome Biol.* **15**, 550 (2014).

29. F. Ramírez, F. Dündar, S. Diehl, B. A. Grüning, T. Manke, deepTools: A flexible platform for exploring deep-sequencing data. *Nucleic Acids Res.* **42**, W187–W191 (2014).

30. A. R. Quinlan, BEDTools: The Swiss-Army tool for genome feature analysis. *Curr. Protoc. Bioinformatics* **47**, 11.12.1–11.12.34 (2014).

31. Y. Zhang, T. Liu, C. A. Meyer, J. Eeckhoute, D. S. Johnson, B. E. Bernstein, C. Nusbaum, R. M. Myers, M. Brown, W. Li, X. S. Liu, Model-based analysis of ChIP-Seq (MACS). *Genome Biol.* **9**, R137 (2008).

32. Q. Wang, M. Li, T. Wu, L. Zhan, L. Li, M. Chen, W. Xie, Z. Xie, E. Hu, S. Xu, G. Yu, Exploring epigenomic datasets by ChIPseeker. *Curr. Protoc.* **2**, e585 (2022).

33. S. Heinz, C. Benner, N. Spann, E. Bertolino, Y. C. Lin, P. Laslo, J. X. Cheng, C. Murre, H. Singh, C. K. Glass, Simple combinations of lineage-determining transcription factors prime cis-regulatory elements required for macrophage and B cell identities. *Mol. Cell* **38**, 576–589 (2010).

34. A. Kiesel, C. Roth, W. Ge, M. Wess, M. Meier, J. Söding, The BaMM web server for de-novo motif discovery and regulatory sequence analysis. *Nucleic Acids Res.* **46**, W215–W220 (2018).

35. A. Dobin, C. A. Davis, F. Schlesinger, J. Drenkow, C. Zaleski, S. Jha, P. Batut, M. Chaisson, T. R. Gingeras, STAR: Ultrafast universal RNA-seq aligner. *Bioinformatics* **29**, 15–21 (2013).

36. G. Perez, G. P. Barber, A. Benet-Pages, J. Casper, H. Clawson, M. Diekhans, C. Fischer, J. N. Gonzalez, A. S. Hinrichs, C. M. Lee, L. R. Nassar, B. J. Raney, M. L. Speir, M. J. van Baren, C. J. Vaske, D. Haussler, W. J. Kent, M. Haeussler, The UCSC Genome Browser database: 2025 Update. *Nucleic Acids Res.* **53**, D1243–D1249 (2025).

37. I. Rauluseviciute, R. Riudavets-Puig, R. Blanc-Mathieu, J. A. Castro-Mondragon, K. Ferenc, V. Kumar, R. B. Lemma, J. Lucas, J. Chèneby, D. Baranasic, A. Khan, O. Fornes, S. Gundersen, M. Johansen, E. Hovig, B. Lenhard, A. Sandelin, W. W. Wasserman, F. Parcy, A. Mathelier, JASPAR 2024: 20th Anniversary of the open-access database of transcription factor binding profiles. *Nucleic Acids Res.* **52**, D174–D182 (2024).

38. C. E. Grant, T. L. Bailey, W. S. Noble, FIMO: Scanning for occurrences of a given motif. *Bioinformatics* **27**, 1017–1018 (2011).

39. A. Liberzon, C. Birger, H. Thorvaldsdóttir, M. Ghandi, J. P. Mesirov, P. Tamayo, The Molecular Signatures Database hallmark gene set collection. *Cell Syst.* **1**, 417–425 (2015).

40. A. Subramanian, P. Tamayo, V. K. Mootha, S. Mukherjee, B. L. Ebert, M. A. Gillette, A. Paulovich, S. L. Pomeroy, T. R. Golub, E. S. Lander, J. P. Mesirov, Gene set enrichment analysis: A knowledge-based approach for interpreting genome-wide expression profiles. *Proc. Natl. Acad. Sci. U.S.A.* **102**, 15545–15550 (2005).

41. L. Berglund, E. Björling, P. Oksvold, L. Fagerberg, A. Asplund, C. A. Szigyarto, A. Persson, J. Ottosson, H. Wernérus, P. Nilsson, E. Lundberg, A. Sivertsson, S. Navani, K. Wester, C. Kampf, S. Hober, F. Pontén, M. Uhlén, A genecentric human protein atlas for expression profiles based on antibodies. *Mol. Cell. Proteomics* **7**, 2019–2027 (2008).
